# Direct demonstration of triplet excimer in purely organic room temperature phosphorescence through rational molecular design

**DOI:** 10.1038/s41377-022-00826-4

**Published:** 2022-05-17

**Authors:** Zhenjiang Liu, Yu Tian, Jie Yang, Aisen Li, Yunsheng Wang, Jia Ren, Manman Fang, Ben Zhong Tang, Zhen Li

**Affiliations:** 1 Institute of Molecular Aggregation Science, Tianjin University, Tianjin, 300072 China; 2 Joint School of National University of Singapore, Tianjin University, International Campus of Tianjin University, Binhai New City, Fuzhou, 350207 China; 3 Shenzhen Institute of Molecular Aggregate Science and Engineering, School of Science and Engineering, The Chinese University of Hong Kong, Shenzhen, Guangdong 518172 China; 4 Tianjin Key Laboratory of Molecular Optoelectronic Sciences, Department of Chemistry, Tianjin University, Tianjin, 300072 China; 5 Department of Chemistry, Wuhan University, Wuhan, 430072 China; 6 Wuhan National Laboratory for Optoelectronics, Huazhong University of Science and Technology, Wuhan, 430074 China

**Keywords:** Optical materials and structures, Optical physics

## Abstract

Organic luminogens with room temperature phosphorescence (RTP) have been paid great attention and developed rapidly for their wide application values. Until now, the internal mechanism and source of phosphorescence are still obscure, especially for the relationship between molecular dimer and RTP emission. Hence, we designed and synthesized eight phenothiazine 5,5-dioxide derivatives to directly reveal how the monomer and dimer in packing affect the RTP behavior. Dimers with strong π-π stacking (*θ* < 20.66°; *d* < 3.86 Å) lead to pure triplet excimer emission, while those with weak π-π stacking (27.02°< *θ* < 40.64°; 3.84 Å < *d* < 4.41 Å) contribute to dual RTP emissions of both monomer and triplet excimer. The valuable information of this work would promote the further development of this research field, as well as others in aggregate.

## Abstract (web summary)

An ideal model containing eight phenothiazine 5,5-dioxide derivatives was established to clearly prove the formation of triplet excimer. Competition between monomer (T_1_) and excimer (T_1_*) based on π-π stacking is the origin for their changed RTP properties.

## Graphical abstract

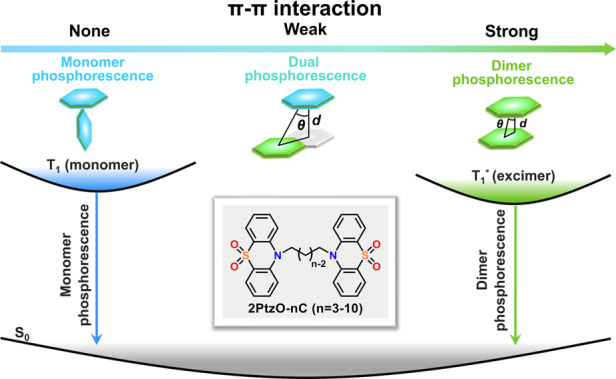

## Introduction

**Fig. 1:**
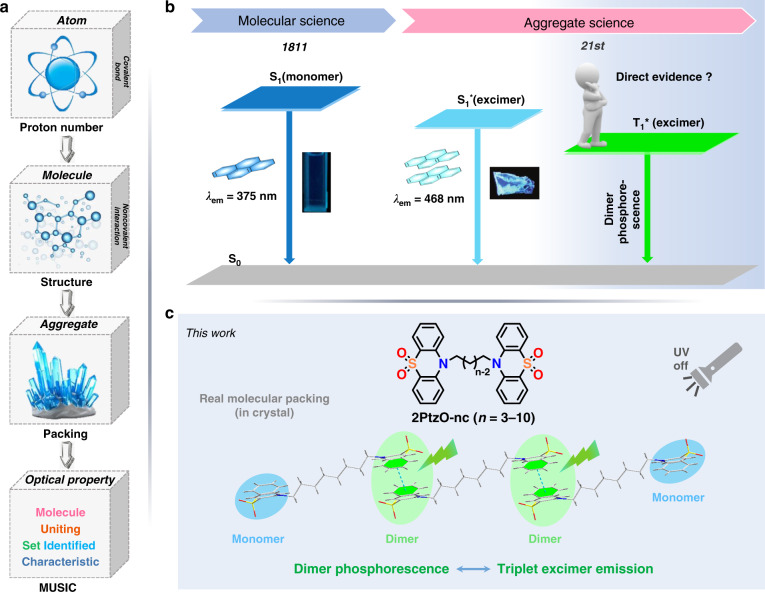
Strategy for molecular design. **a** Critical factor affecting chemical properties: Atoms with different proton numbers form molecule through covalent bonds, while molecules with different or the same structure form molecular aggregates through non-covalent bonds, lead to the characteristic of Molecular Uniting Set Identified Characteristic (MUSIC). **b** Development process from “*molecular science*” to “*aggregate science*”. **c** Different room temperature phosphorescence (RTP) emissions from monomer and dimer based on different molecular packing of phenothiazine-5,5-dioxide derivatives

Advance and development in organic optoelectronic materials have enabled excellent innovations in our daily life for their wide applications in organic light emitting diodes (OLEDs), organic field effect transistors (OFETs), solar cells and bio/chemo probing etc^1–5^. In order to design and produce more smart optoelectronic materials, it is particularly important to make clear their internal mechanism, especially for the relationship between material structure and properties. Throughout the history, people’s perception of it is changing all the time (Fig. 1a). Since 1803, the atomism was proposed by Dalton, in which the proton number was considered to determine the species and properties of a substance^6^. Although the atomism could explain some chemical phenomena, the defects were objective. Accordingly, the molecular hypothesis was put forward by Avogadro in 1811^7^. After atoms forming molecules through covalent bonding, the molecular structure plays the key role in the properties of individual molecule, as it determines the energy level characteristics. Thus, the change of molecular structure is the most direct way to adjust the corresponding optoelectronic property^8,9^. However, there are still some photophysical phenomena that couldn’t be well understood by molecular science^10,11^. Particularly, scientists found many materials show distinct properties in the forms of different aggregates, for example, polymorphism, which could be termed as “Molecular Uniting Set Identified Characteristic (MUSIC)”, that is the basic atoms correspond to notes, and a melody with alignment of notes is similar to molecules constructed by atoms with specific sequences. Correspondingly, the MUSICs, which are heavily dependent on the aggregated states with various packing modes, resemble a symphony with the coming together of music produced by different instruments^12^. Thus, in the 21st century, scientists turned their eyes to the effect of molecular stacking, that is molecular aggregation science, as most materials were utilized in solid or aggregate states^10–18^.

As one of the typical organic optoelectronic materials, luminogens show heavy relationship and great sensitivity to molecular stacking. It was found that the change between solution and aggregate or different aggregate states could often lead to the much different luminescent properties^19^. For example, excimer, the short-lived dimeric molecule formed from two species (Fig. 1b), often leads to the much red-shifted emission from solution to solid state^20^. Related single crystal structures indicate that molecular dimer with π-π interaction has played significant role in emission, especially excimer emission. Upon excitation, a molecule in excited state will approach the adjacent one in ground state through attraction, then lead to the formation of excimer with lowered potential energy curve and narrowed energy gap. Based on this point, organic luminogens should be good platform to explore the relationship between molecular packing and property.

Similar case is considered for purely organic luminogens with room temperature phosphorescence (RTP), in which the molecular packing affects the RTP effect heavily^21–37^. Particularly, molecular dimer, the most important intermolecular interaction form, has been explored frequently, such as dimer with H-aggregation, dimer with intermolecular hydrogen bond, dimer with intermolecular halogen bond and dimer with n-π or π-π interaction etc (Chart S1). Then what about the exact role of molecular dimer in RTP effect? As excimer was easy to be formed within molecular dimer, was the RTP also from triplet excimer? To be exact, an ideal model is urgently needed to reveal the generation process and luminescence behavior of dimer-related room temperature phosphorescence.

In our previous work, phenothiazine-5,5-dioxide group with planar conformation has been found to easily form intermolecular π-π interaction in solid state, which then contributed much to the persistent RTP effect^24,38^. Inspired by it, in this work, a rational molecular design was carried out and eight target compounds (**2PtzO-nC**, *n* = 3–10) with two phenothiazine-5,5-dioxide groups linked by alkyl chains with different carbon numbers were synthesized accordingly (Fig. 1c). For these compounds, the two phenothiazine-5,5-dioxide groups acted as two independent RTP cores and non-conjugated alkyl chains were introduced to adjust the molecular packing without changing the electronic structure of single molecules. With this kind of molecular structure, the monomer and dimer based RTP emissions could both appear in one compound and be distinguished easily, then being beneficial a lot to the clarification of the role of molecular dimer in RTP emission. Just as expected, the molecular dimers with different strengths of intermolecular π-π interaction have been formed in crystal state for these compounds. Among them, the crystals with both bilateral dimers involving strong π-π stacking show pure triplet excimer emission, while those with just unilateral dimer in strong π-π stacking or both bilateral dimers in weak π-π stacking gave dual RTP emissions from monomer and triplet excimer. Thus, the direct demonstration of triplet excimer in purely organic room temperature phosphorescence was realized, to provide a unique platform for the investigation of inherent mechanism.

## Results

### Molecular preparation and characterizations

Eight target compounds (**2PtzO-nC**, *n* = 3–10) were easily synthesized in two steps with C-N coupling following an oxidation reaction in the presence of hydrogen peroxide (Scheme S1). The resultant compounds have been well characterized by ^1^H and ^13^C NMR, high-revolution mass spectra (HRMS) and high-performance liquid chromatogram (HPLC) spectra (Fig. S1) to certify their chemical structure and purity. Then, thermo-gravimetric analysis (TGA) and differential scanning calorimeter (DSC) measurements were carried out to evaluate their thermal stability (Fig. S2). It was found that no obvious glass transition happened in the heating process and their thermal decomposition temperatures ranged from 389 to 434 °C, indicating the good thermal stability. Besides, the melting points of **2PtzO-7C** and **2PtzO-8C** were found to be higher than that of **2PtzO-6**C with shorter alkyl chain. This indicates the more rigid environment in **2PtzO-7C** and **2PtzO-8C** crystals, which will be much beneficial to their corresponding RTP emission.

### Photophysical properties in solution and solid states

**Fig. 2:**
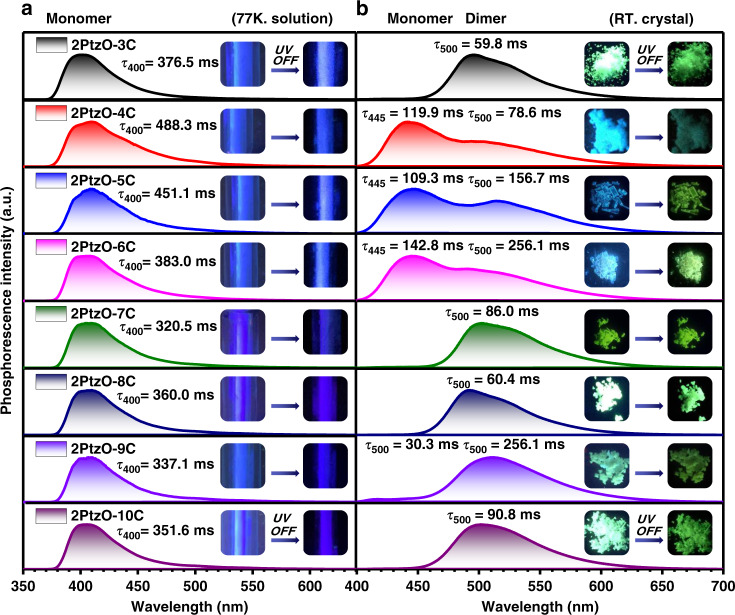
Phosphorescence property of 2PtzO-nC in different states. **a** The phosphorescence spectra of eight compounds (**2PtzO-nC**) in dilute DCM solution (10 µM; λ_ex_ = 330 nm) at 77 K and **b** in crystal state at room temperature (λ_ex_ = 365 nm). Insert graph: photographs of **2PtzO-nC** in corresponding states before and after the removal of UV irradiation

Then their photophysical properties were studied in solution and solid states (Tables S1 and S2). Compounds **2PtzO-nC** have similar UV-vis absorption bands at around 273, 300 and 336 nm in dilute dichloromethane (DCM) solution (Fig. S3a), indicating the introduction of non-conjugated alkyl chain has little effect on the energy level of chromophore. In solid state, little red-shifted UV-vis absorptions can be observed for these compounds, indicating the formation of J-aggregation (Fig. S3b). As we all known, in a dilute solution, the solute molecules are uniformly dispersed in the solvent, and the interaction between the molecules is limited. Therefore, the luminescence behavior in solution is attributed to monomers. At room temperature, there is just one fluorescence peak at around 360 nm in DCM solution, while a phosphorescence with blue afterglow appears at 77 K for the rigidification of environment (Fig. S4a and b). Further on, the phosphorescence spectra and corresponding lifetimes in solutions at 77 K were measured to verify their monomer phosphorescence characteristics (Fig. S4c and d). As shown in Fig. 2, all of the eight target compounds show similar phosphorescence profile in monomer state (@ 77 K) with phosphorescence peaks at about 400 nm. In addition, the phosphorescence lifetimes in DCM solution at 77 K are also less different from each other, ranging from 320.5 to 488.3 ms. When the eight target compounds are doped into polymethyl methacrylate (PMMA) film, phosphorescence is not observed at room temperature, while the films exhibit almost the same blue phosphorescence at 77 K with lifetimes ranging from 343.1 ms to 375.0 ms (Fig. S5). All of these experimental results well demonstrate the similar electronic property in monomer state for these target compounds, regardless of the different alkyl chain lengths in molecular structures.

When it turned to solid state, much different phosphorescence behaviors were observed for these compounds. To well match the RTP property and molecular packing, their single crystals were cultured by slow solvent evaporation and the corresponding photoluminescence (PL) behaviors, especially for the RTP behaviors, were studied in detail (Figs. S6–S8 and Fig. 2). Upon the exposure of a 365 nm UV lamp, **2PtzO-nC** crystals show mainly two different kinds of emission colors: green for **2PtzO-3C**/**2PtzO-7C**/**2PtzO-8C**/**2PtzO-9C**/**2PtzO-10C**, blue for **2PtzO-4C**/**2PtzO-5C**/**2PtzO-6C**. After stopping the photo-excitation, also different kinds of RTP behaviors could be observed (Fig. 2 and Fig. S9). In crystals **2PtzO-3C**/**2PtzO-7C**/**2PtzO-8C**/**2PtzO-10C**, just one RTP emission peak at about 500 nm could be detected, while a tiny one at about 445 nm appeared in **2PtzO-9C**. As for **2PtzO-4C/2PtzO-5C/2PtzO-6C**, two distinct RTP emission peaks at about 445 and 500 nm were presented. Among them, the RTP peak at 445 nm should be derived from the red-shifted phosphorescence of monomer (400 nm) in solution state at 77 K, while the one at 500 nm was thought to be from the effect of molecular packing, such as molecular dimer with triplet excimer emission. Also, much different RTP lifetimes were obtained for the crystals of these eight compounds. For the RTP peak at 445 nm, the corresponding lifetimes ranged from 30.3 ms to 142.8 ms, while those at 500 nm were from 59.8 ms to 256.1 ms. As the lifetimes for the bands at 445 nm are comparable to or even longer than those at 500 nm, the phosphorescence emission from high-lying triplet excited state (i.e. T_2_) can be excluded^39,40^. Based on the different RTP behaviors of these compounds, the application of multiple anti-counterfeiting was successfully realized (Fig. S10). In comparison with the solution state at 77 K, it is not hard to find that molecular packing, rather than electronic property of single molecule, should be mainly responsible for their different RTP properties.

### Analyses of single-crystal structure

**Fig. 3:**
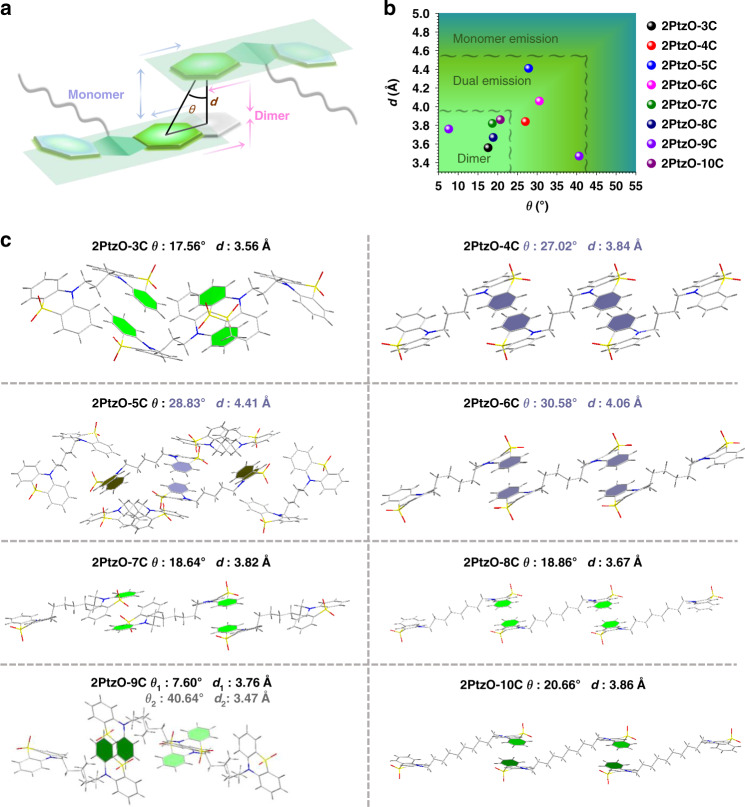
X-ray single crystal diffraction data of 2PtzO-nC crystals. **a** Cartoon graph of π-π stacking: the movements of magenta arrows represent stronger π-π interaction and blue arrows represent weaker π-π interaction (*d*: vertical distance between the adjacent benzene planes. *θ*: displacement angle, the angle between the centroid–centroid line and the vertical line). **b** The scatter diagram based on the vertical distance (*d*), displacement angle (*θ*) and RTP behavior of **2PtzO-nC** crystals. **c** Single-crystal structures of the eight target compounds. Phenyl rings involved in the stronger π-π interactions are labeled by green color, weaker π-π interactions by indigo blue color and none π–π interactions by dark yellow color

In order to clarify the relationship between RTP emission and molecular packing, the single crystal structures for these eight compounds were measured and analyzed carefully (Tables S3 and S4). Figure 3 and Figs. S11–S14 show the local and entire packing modes of **2PtzO-nC** crystals. It could be clearly observed that intermolecular π-π interactions widely exist for these crystals, although the corresponding strengths are different from each other for the introduction of different lengths of alkyl chain between two phenothiazine 5,5-dioxide units. To well evaluate the strength of π-π interactions, the analyses of displacement angle (*θ*) and vertical distance (*d*) for the adjacent benzene rings involved in π–π stacking were carried out^41^, in which smaller displacement angle and shorter vertical distance indicate stronger π–π interaction (Fig. 3a). As shown in Figs. 2, 3c, for crystals **2PtzO-3C/2PtzO-7C**/**2PtzO-8C**/**2PtzO-10C** with pure triplet excimer emission (@500 nm), strong π–π interaction could be clearly observed for the bilateral molecular dimers with small displacement angles (17.56° < *θ* < 20.66°) and short vertical distances (3.56 Å < *d* < 3.86 Å). In crystal **2PtzO-9C** with dominant triplet excimer emission (@500 nm) and weak monomer RTP one (@445 nm), two kinds of π–π interaction existed for the bilateral molecular dimers, in which one was strong (*θ* = 7.60°, *d* = 3.76 Å) and the other was relatively weak (*θ* = 40.64°, *d* = 3.47 Å). As for other three crystals with comparable dual RTP emissions, much weaker π–π interactions were presented. For example, the displacement angles (*θ*) and vertical distances (*d*) for **2PtzO-4C** and **2PtzO-6C** increased to 27.02/30.58° and 3.84/4.06 Å for the bilateral molecular dimers. In **2PtzO-5C**, just unilateral dimer has been formed with weak π–π interaction (*θ* = 27.83°, *d* = 4.41 Å). These weak π–π interactions within molecular dimers will lead to the competition between monomer and excimer phosphorescence, thus resulting in the dual RTP emissions. All in all, the universality of the relationship between π–π stacking strength and RTP behavior was clearly and accurately summarized in Fig. 3b. The smaller the displacement angle (*θ*) and the shorter the vertical distance (*d*), the stronger the π–π interaction in molecular dimer, and the greater the phosphorescence of the triplet excimer.

Further on, time-dependent density functional theory (TD-DFT) calculations were carried out to study the relationship between π-π stacking and RTP emission. In particular, the natural transition orbitals (NTOs) of T_1_ state were calculated for the molecular dimer (Figs. S15–S18). As it is easily seen, intermolecular orbital couplings for T_1_ state widely exist in the molecular dimers with strong π-π interactions, such as **2PtzO-3C**/**2PtzO-7C**/**2PtzO-8C**/**2PtzO-10C** crystals, which would be much beneficial to their pure triplet excimer emission. Interestingly, for the weaker dimer in **2PtzO-9C** crystal, although the displacement angle (*θ* = 40.64°) is relatively large, the short vertical distance (*d* = 3.47 Å) still leads to the weak orbital coupling for T_1_ state. This should be the main reason for its dominant triplet excimer emission in crystal state. As for **2PtzO-4C**/**2PtzO-5C**/**2PtzO-6C** crystals with comparable dual RTP emissions, no obvious orbital coupling in T_1_ state could be observed for the molecular dimer with weak π–π stacking. Thus, the strong π–π stacking in dimer as the main origin for triplet excimer emission could be further demonstrated. In addition, the calculations of HOMO/LUMO orbital distributions for these molecular dimers were calculated. As shown in the Figs. S19–S26, obvious electron cloud communications could be observed for the molecular dimers with π-π interactions in LUMO orbital distributions. This would affect the corresponding luminescent behaviors, such as the triplet excimer emission.

### Excited process of RTP emission

**Fig. 4:**
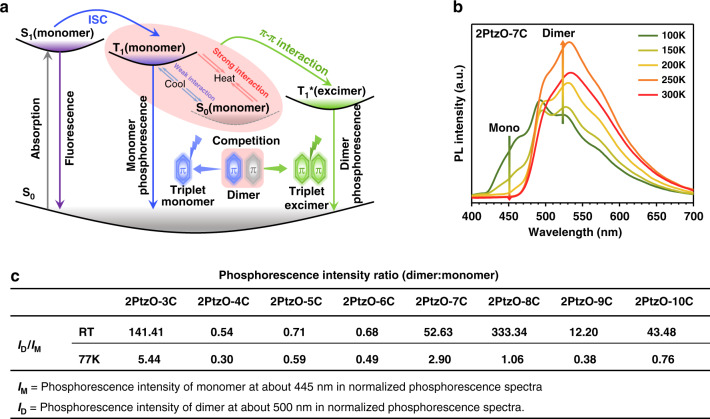
RTP emission mechanism. **a** The proposed mechanism for monomer and dimer based RTP emissions: pure excimer RTP emission exists in the dimer with strong π-π interaction and monomer-excimer dual RTP emissions exist in the dimer with weak π-π interaction respectively. **b** The PL spectra of **2PtzO-7C** crystal at different temperatures. **c** The ratios of dimer to monomer phosphorescent intensity of **2PtzO-nC** crystals at room temperature and 77 K

According to the relationship between molecular packing and RTP property, a rational excited process was proposed. As depicted in Fig. 4a, upon excitation, the monomers in ground state (S_0_) are first excited to the first excited singlet state (S_1_), then some of them will return to S_0_ through fluorescence emission, while some others can jump to the first excited triplet state (T_1_) through intersystem crossing (ISC) transition. Because of the formation of molecular dimer with π-π interaction, the excitons in T_1_ state will tend to approach the adjacent one in S_0_ state to form triplet excimer (T_1_^*^). At this time, for the dimers with strong π-π interaction, the formation of triplet excimer (T_1_^*^) should be much easier and faster, thus leading to the pure triplet excimer emission. As for the ones with weak π-π interaction, the competition between monomer (T_1_) and excimer (T_1_^*^) phosphorescence could occur, thus resulting in the dual RTP emissions.

As the formation of triplet excimer is a typical kinetic process, it should be affected by temperature^42^. Consequently, the temperature-dependent phosphorescence spectra from 100 to 300 K were measured for these eight crystals (Figs. S27 and S28). Unlike those at room temperature, the monomer phosphorescence emission at about 445 nm existed for all of them at low temperature, since the formation of excimer was largely restricted at this time. Taking **2PtzO-7C** crystal as example, the dual phosphorescence emissions were presented at 100 K, in which the shoulder peak at about 445 nm should be attributed to monomer and the main one at about 500 nm was from molecular dimer (Fig. 4b). With the increase of temperature from 100 to 250 K, the dimer emission gradually increased for the promoted molecular motion of excited monomer towards ground one to form excimer. At the same time, the monomer phosphorescence emission was gradually weakened and nearly disappeared at 250 K for the failure of competition with excimer one. If further increasing temperature, the triplet excimer emission would be decreased for the excessive thermal motion of molecules. Similar phenomenon could also be observed for **2PtzO-3C** crystal (Fig. S27). As for other six crystals, thermodynamic process was dominant for the triplet excimer. Therefore, the dimer based RTP emission at about 500 nm always decreased with the rise of temperature from 100 to 350 K for the enhanced non-radiative motion (Figs. S27 and S28). During this process, the competition between monomer and excimer phosphorescence could be clearly observed for all of them, in which low temperature was beneficial to monomer phosphorescence and higher one did the opposite. Just as summarized in Fig. 4c, the phosphorescence intensity ratios of excimer to monomer at room temperature were always larger than those at low temperature (i.e., 77 K) (Figs. S29–S31 and Tables S5 and S6), which further illustrated the reliability of the competition process between monomer (T_1_) and triplet excimer (T_1_*) in Fig. 4a. Based on these temperature-dependent phosphorescence behaviors, the formation of triplet excimer in the RTP process could be further certified. As it involves the movement of one molecule in T_1_ state to another one in ground state, the triplet excimer should belong to dynamic excimer. Besides, because of the short lifetime of singlet excitons, the kinetic process for the formation of singlet excimer was hard to be achieved, thus no corresponding singlet excimer emission could be observed for these target compounds.

Correspondingly, the phosphorescence processes in different states and different compounds could be simplified as two ways below:

(a) S_0_(monomer) →S_1_(monomer)→T_1_(monomer)→S_0_

(b) S_0_(monomer)→S_1_(monomer)→T_1_(monomer)→T_1_ + S_0_(dimer)

→T_1_*(excimer)→ S_0_

Phosphorescence of **2PtzO-nC** solution at 77 K goes through way (a); **2PtzO-4C**, **2PtzO-5C**, **2PtzO-6C** and **2PtzO-9C** crystals go through both ways (a) and (b), while **2PtzO-3C**, **2PtzO-7C**, **2PtzO-8** and **2PtzO-10C** crystals were way (b). Thus, through simply alkyl chain regulation, the adjustment of excited process in solid state was successfully realized for the changed molecular packing.

## Conclusions

An ideal model containing eight phenothiazine 5,5-dioxide derivatives was established to clearly and accurately prove the formation of triplet excimer. Among them, **2PtzO-3C**, **2PtzO-7C**, **2PtzO-8C**, **2PtzO-10C** crystals only exhibit pure excimer RTP emission while other crystals show dual RTP emissions of both monomer and excimer. Detailed analyses of their single crystals demonstrated that the pure excimer RTP results from strong π–π interaction (*θ* < 20.66°; *d* < 3.86 Å), while the dual RTP emissions come from weak one (27.02°< *θ* < 40.64; 3.84 Å < *d* < 4.41 Å). Further on, the corresponding excited processes of RTP emission were successfully proposed with the aid of temperature-dependent phosphorescence measurements for these eight crystals. It was found that the competition between monomer (T_1_) and excimer (T_1_*) should be the main origin for their changed RTP properties. It is believed that this work would be of great importance for gaining a clear and deep understanding of the whole RTP process, thus guiding the further development of this research area, as well as others in aggregate^43–51^.

## Materials and methods

### Syntheses

#### 2PtzO-3C

To an ice-cooled suspension of NaH (60% in mineral oil, 0.6 g, 15 mmol) in dry *N*,*N*-Dimethylformamide (DMF) solution (25 mL) was added phenothiazine (2 g, 10 mmol), and stirred for 30 min under N_2_. Then, 1,3-dibromopropane (1.2 mL, 12 mmol) was added dropwise and the mixture was stirred at 0 °C under N_2_. After 4 h, water was added to the mixture to quench the reaction. The organic layer was collected with dichloromethane (DCM) and dried over by anhydrous Na_2_SO_4_ and concentrated by rotary evaporation. The crude product was purified by column chromatography on silica gel using petroleum ether (PE)/DCM (10: 1 v/v) as eluent and to afford a white solid. Then the collected product was dissolved in DCM (20 mL), acetic acid (9 mL) and H_2_O_2_ (6 mL). After reacting for another 24 h at 60 °C, the reaction mixture was extracted with dichloromethane and further purified by column chromatography using PE/DCM (1:5 v/v) as eluent to afford a white solid in a yield of 27.4%. mp: 325 °C; ^1^H NMR (400 MHz, CDCl_3_): *δ* 7.98 (dd, J_1_ = 9.6 Hz, J_2_ = 1.6 Hz, 4H), 7.31 (ddd, J_1_ = 8.6 Hz, J_2_ = 1.6 Hz, 4H), 7.21-7.12 (m, 8H), 4.27 (t, J = 5.2 Hz, 4H), 2.14-2.09 (m, 2H); ^13^C NMR (100 MHz, CDCl_3_): *δ* 141.50, 133.01, 125.70, 122.93, 122.19, 117.34, 41.97, 25.50; HRMS (ESI), m/z: [M + Na]^+^ calcd. for C_27_H_22_N_2_NaO_4_S_2_, 525.0913; found, 525.0935.

#### 2PtzO-4C

White solid (88.4%). mp: 301.3 °C; ^1^H NMR (400 MHz, CDCl_3_): *δ* 8.07 (dd, J_1_ = 10 Hz, J_2_ = 2 Hz, 4H), 7.56-7.52 (m, 4H), 7.30-7.22 (m, 8H), 4.16 (t, J = 6.2 Hz, 4H), 1.92-1.89 (m, 4H); ^13^C NMR (100 MHz, CDCl_3_): *δ* 141.42, 133.20, 125.41, 123.59, 122.22, 116.77, 67.18, 23.76; HRMS (ESI), m/z: [M + Na]^+^ calcd. for C_28_H_24_N_2_NaO_4_S_2_, 539.1070; found, 539.1074.

#### 2PtzO-5C

White solid (68.7%). mp: 249.2 °C; ^1^H NMR (400 MHz, CDCl_3_): *δ* 8.12 (d, J = 8 Hz, 4H), 7.56 (t, J = 8 Hz, 4H), 7.29 (d, J = 8.4 Hz, 8H), 4.17 (t, J = 7 Hz, 4H), 1.90-1.83 (m, 4H), 1.53-1.45 (m, 2H); ^13^C NMR (100 MHz, CDCl_3_): *δ* 141.17, 132.20, 124.92, 123.61, 122.01, 47.18, 26.21, 22.68; HRMS (ESI), m/z: [M + Na]^+^ calcd. for C_29_H_26_N_2_NaO_4_S_2_, 553.1226; found, 553.1248.

#### 2PtzO-6C

White solid (66.7%). mp: 236.6 °C; ^1^H NMR (400 MHz, CDCl_3_): *δ* 8.10 (dd, J_1_ = 9.6 Hz, J_2_ = 1.6 Hz, 4H), 7.58 (ddd, J_1_ = 8.8 Hz, J_2_ = 1.6 Hz, 4H), 7.32-7.24 (m, 8H), 4.16 (t, J = 7.4 Hz, 4H), 1.90-1.85 (m, 4H), 1.45-1.41 (m, 4H); ^13^C NMR (100 MHz, CDCl_3_): *δ* 141.14, 133.17, 124.77, 123.67, 121.96, 116.37, 47.69, 26.8, 25.99; HRMS (ESI), m/z: [M + Na]^+^ calcd. for C_30_H_28_N_2_NaO_4_S_2_, 567.1383; found, 567.1361.

#### 2PtzO-7C

White solid (44.5%). mp: 263.2 °C; ^1^H NMR (400 MHz, CDCl_3_): *δ* 8.09 (d, J = 8 Hz, 4H), 7.59 (t, J = 7.8 Hz, 4H), 7.31-7.23 (m, 8H), 4.13 (t, J = 7.2 Hz, 4H), 1.88-1.81 (m, 4H), 1.44-1.36 (m, 4H), 1.27-1.22 (m, 2H); ^13^C NMR (100 MHz, CDCl_3_): *δ* 141.08, 133.17, 124.63, 123.71, 121.93, 116.26, 48.03, 28.80, 26.82, 26.43; HRMS (ESI), m/z: [M + Na]^+^ calcd. for C_31_H_30_N_2_NaO_4_S_2_, 581.1539; found, 581.1536.

#### 2PtzO-8C

White solid (47.2%). mp: 244.4 °C; ^1^H NMR (400 MHz, CDCl_3_): *δ* 8.10 (dd, J_1_ = 9.2 Hz, J_2_ = 1.6 Hz, 4H), 7.60 (t, J = 8 Hz, 4H), 7.36-7.24 (m, 8H), 4.15 (t, J = 7.4 Hz, 4H), 1.90-1.83 (m, 4H), 1.43-1.36 (m, 8H); ^13^C NMR (100 MHz, CDCl_3_): *δ* 140.99, 133.05, 124.55, 123.65, 121.81, 116.12, 48.06, 28.92, 26.78, 26.23; HRMS (ESI), m/z: [M + Na]^+^ calcd. for C_32_H_32_N_2_NaO_4_S_2_, 595.1696; found, 595.1686.

#### 2PtzO-9C

White solid (80.0%). mp: 186.4 °C; ^1^H NMR (400 MHz, CDCl_3_): *δ* 8.09 (dd, J_1_ = 9.2 Hz, J_2_ = 1.6 Hz, 4H), 7.59 (ddd, J_1_ = 8.6 Hz, J_2_ = 1.6 Hz, 4H), 7.32-7.22 (m, 8H), 4.13 (t, J = 7.6 Hz, 4H), 1.90-1.83 (m, 4H), 1.40-1.31 (m, 4H), 1.30-1.22 (m, 6H); ^13^C NMR (100 MHz, CDCl_3_): *δ* 141.01, 133.18, 124.47, 123.74, 121.89, 116.16, 48.27, 29.37, 28.93, 26.86, 26.51; HRMS (ESI), m/z: [M + Na]^+^ calcd. for C_33_H_34_N_2_NaO_4_S_2_, 609.1852; found, 609.1857.

#### 2PtzO-10C

White solid (82.1%). mp: 216.3 °C; ^1^H NMR (400 MHz, CDCl_3_): *δ* 8.10 (dd, J_1_ = 9.2 Hz, J_2_ = 1.6 Hz, 4H), 7.60 (ddd, J_1_ = 8.8 Hz, J_2_ = 1.6 Hz, 4H), 7.33-7.23 (m, 8H), 4.14 (t, J = 7.8 Hz, 4H), 1.92-1.85 (m, 4H), 1.46-1.40 (m, 4H), 1.37-1.30 (m, 8H). ^13^C NMR (100 MHz, CDCl_3_) *δ* (ppm): 140.98, 133.18, 124.41, 123.75, 121.87, 113.09, 48.39, 29.41, 29.19, 26.88, 26.68; MS (ESI), m/z: [M + Na]^+^ calcd. for C_34_H_36_N_2_NaO_4_S_2_, 623.2009; found, 623.2005.

## Methods

^1^H NMR spectra and ^13^C NMR spectra were recorded on a 400 MHz Bruker AVANCE III spectrometer using CDCl_3_ as solvent. Mass spectra were measured on a UHPLC/Q-TOF MS spectrophotometer. High-performance liquid chromatogram spectra were recorded on Agilent 1100 HPLC. Thermo-gravimetric analysis curves and differential scanning calorimeter curves were recorded on Thermo Gravimetric Analysis TG-209F3 and Differential Scanning Calorimeter DSC214 ployma. UV-vis spectra were measured on a Shimadzu UV-2600. Photoluminescence spectra and phosphorescence lifetimes were performed on a Hitachi F-4600 fluorescence spectrophotometer. Photoluminescence quantum yields, fluorescence lifetimes and temperature-dependent phosphorescence spectra were determined with FLS1000 spectrometer. The single-crystal X-ray diffraction data of these samples were collected in XtaLAB SuperNova X-ray diffractometer.

The Gaussian 09 program was utilized to perform the TD-DFT calculations. The ground state (S_0_) geometries of dimers were obtained from the single crystal structures and no further geometry optimization was conducted in order to maintain the specific molecular configurations and corresponding intermolecular locations. The HOMO/LUMO orbital distributions and natural transition orbitals (NTOs) of T_1_ state of dimers were evaluated by the TD-m062x/6-31 g*.

## Supplementary information

Supplementary Information

Article confidentiality and copyright transfer agreement

Copyrright for Chart S1-1

Copyrright for Chart S1-2

Copyrright for Chart S1-3

Copyrright for Chart S1-4

Copyrright for Chart S1-5

## Data Availability

The authors declare that the data supporting the findings of this study are provided in the Information file. All data are available from the authors upon request.
