# Supplementary material for: Direct demonstration of triplet excimer in purely organic room temperature phosphorescence through rational molecular design
Source: Light Sci Appl. 2022 May 17;11:142. doi: 10.1038/s41377-022-00826-4 (PMC9114335; doi:10.1038/s41377-022-00826-4)
Supplement: Supplementary file 1 — Supplementary Information [file 41377_2022_826_MOESM1_ESM.pdf]

Supplementary Information for

Direct demonstration of triplet excimer in purely organic room temperature phosphorescence through rational molecular design

Zhenjiang Liu[1], Yu Tian[1], Jie Yang[1]*, Aisen Li[5], Yunsheng Wang[1], Jia Ren[1], Manman Fang[1], Ben Zhong Tang[1,3]*, Zhen Li[1,2,4,5,6]*

[1] Institute of Molecular Aggregation Science, Tianjin University, Tianjin, 300072, China.

[2] Tianjin Key Laboratory of Molecular Optoelectronic Sciences, Department of Chemistry, Tianjin University, Tianjin, 300072, China.

[3] Shenzhen Institute of Molecular Aggregate Science and Engineering, School of Science and Engineering, The Chinese University of Hong Kong, Shenzhen, Guangdong, 518172, China.

[4] Department of Chemistry, Wuhan University, Wuhan, 430072, China.

[5] Joint School of National University of Singapore, Tianjin University, International Campus of Tianjin University, Binhai New City, Fuzhou, 350207, China.

[6] Wuhan National Laboratory for Optoelectronics, Huazhong University of Science and Technology, Wuhan 430074, China.

This PDF file includes:

Chart S1 Molecular structures and the packing modes in dimer of the reported persistent RTP materials.
Scheme S1 The synthetic route of **2PtzO-nC**.
Fig. S1 High performance liquid chromatography diagrams of **2PtzO-nC**.
Fig. S2 a Thermo-gravimetric Analysis (TGA) curves. **b** Differential scanning calorimeter (DSC) curves of **2PtzO-nC**.
Table S1 Photophysical data of **2PtzO-nC** (n = 3-6) in solutions and crystals.
Table S2 Photophysical data of **2PtzO-nC** (n = 7-10) in solutions and crystals.
Fig. S3 a The UV-vis absorption spectra of **2PtzO-nC** in the dilute DCM solution (10 μM) and (**b**) in solid state.
Fig. S4 a The steady-state PL spectra of **2PtzO-nC** in dilute DCM solution (10 μM) at room temperature and **b**) at 77K (λ_{ex} = 330 nm). **c** The phosphorescence spectra of **2PtzO-nC** in dilute DCM solution (10 μM) at 77K (λ_{ex} = 330 nm). **d** The phosphorescence-decay curves of **2PtzO-nC** in dilute DCM solution (10 μM) at 77K (λ_{ex} = 330 nm).
Fig. S5 a The phosphorescence spectra of **2PtzO-nC** doped in polymethyl methacrylate (PMMA) matrix with mass ratio of 1% at room temperture (λ_{ex} = 365 nm); **b** The phosphorescence spectra of **2PtzO-nC** doped in polymethyl methacrylate (PMMA) matrix with mass ratio of 1% at 77K (λ_{ex} = 330 nm). **c** Time-resolved phosphorescence-decay curves of **2PtzO-nC** in polymethyl methacrylate (PMMA) matrix at 77K (@ 405 nm; λ_{ex} = 330 nm).
Fig. S6 a The steady-state PL spectra of **2PtzO-nC** in crystal state at room temperature (λ_{ex} = 330 nm). **b** PL decay curves of **2PtzO-nC** (n = 3-6) in crystal state (λ_{ex} = 330 nm; monomer fluorescence). **c** PL decay curves of **2PtzO-nC** (n = 7-10) in crystal state (λ_{ex} = 330 nm; monomer fluorescence).
Fig. S7 a The excitation spectra of **2PtzO-nC** in crystal state for the phosphorescence band at 445 nm and **b** at 500 nm.
Fig. S8 a Time-resolved RTP-decay curves of **2PtzO-nC** crystals at 445 nm (λ_{ex} = 365 nm; monomer phosphorescence). **b** Time-resolved RTP-decay curves of **2PtzO-nC** (n = 3-6) crystals at 500 nm (λ_{ex} = 365 nm; dimer phosphorescence). **c** Time-resolved RTP-decay curves of **2PtzO-nC** (n = 7-10) crystals at 500 nm (λ_{ex} = 365 nm; dimer phosphorescence).
Fig. S9 Photographs of **2PtzO-nC** crystals taken at different times before and after removing the excitation of a 365 nm UV lamp.
Fig. S10 Application of multiple anti-counterfeiting.
Fig. S11 Entire and local packing modes of **2PtzO-3C** and **2PtzO-4C**.
Fig. S12 Entire and local packing modes of **2PtzO-5C** and **2PtzO-6C**.
Fig. S13 Entire and local packing modes of **2PtzO-7C** and **2PtzO-8C**.
Fig. S14 Entire and local packing modes of **2PtzO-9C** and **2PtzO-10C**.
Table S3 Structure data of single crystals of **2PtzO-nC** (n = 3-6).
Table S4 Structure data of single crystals of **2PtzO-nC** (n = 7-10).
Fig. S15 Natural transition orbitals (NTOs) of the T_1 state for the dimers of **2PtzO-3C** and **2PtzO-4C**.
Fig. S16 Natural transition orbitals (NTOs) of T_1 state for the dimers of **2PtzO-5C** and **2PtzO-6C**.

Fig. S17 Natural transition orbitals (NTOs) of T_1 state for the dimers of **2PtzO-7C** and **2PtzO-8C**.

Fig. S18 Natural transition orbitals (NTOs) of T_1 state for the dimers of **2PtzO-9C** and **2PtzO-10C**.

Fig. S19 The HOMO, LUMO orbital distributions for the dimer unit of **2PtzO-3C**.

Fig. S20 The HOMO, LUMO orbital distributions for the dimer unit of **2PtzO-4C**.

Fig. S21 The HOMO, LUMO orbital distributions for the dimer unit of **2PtzO-5C**.

Fig. S22 The HOMO, LUMO orbital distributions for the dimer unit of **2PtzO-6C**.

Fig. S23 The HOMO, LUMO orbital distributions for the dimer unit of **2PtzO-7C**.

Fig. S24 The HOMO, LUMO orbital distributions for the dimer unit of **2PtzO-8C**.

Fig. S25 The HOMO, LUMO orbital distributions for the dimer units of **2PtzO-9C**.

Fig. S26 The HOMO, LUMO orbital distributions for the dimer unit of **2PtzO-10C**.

Fig. S27 The PL spectra of **2PtzO-nC** (n = 3-6) crystals at different temperatures.

Fig. S28 The PL spectra of **2PtzO-nC** (n = 7-10) crystals at different temperatures.

Fig. S39 The normalized phosphorescence spectra of **2PtzO-nC** (n = 3-6) crystals at room temperature and 77 K.

Fig. S30 The normalized phosphorescence spectra of **2PtzO-nC** (n = 7-10) crystals at room temperature and 77 K.

Fig. S31 **a** Time-resolved PL-decay curves for monomer phosphorescence (@445 nm) of **2PtzO-nC** crystals at 77 K. **b** Time-resolved PL-decay curves for dimer phosphorescence (@500 nm) of **2PtzO-nC** crystals at 77 K.

Table S5 The phosphorescence intensity and ratio of dimer to monomer for **2PtzO-nC** (n = 3-6) crystals at 77 K and room temperature.

Table S6 The phosphorescence intensity and ratio of dimer to monomer for **2PtzO-nC** (n = 7-10) crystals at 77 K and room temperature.

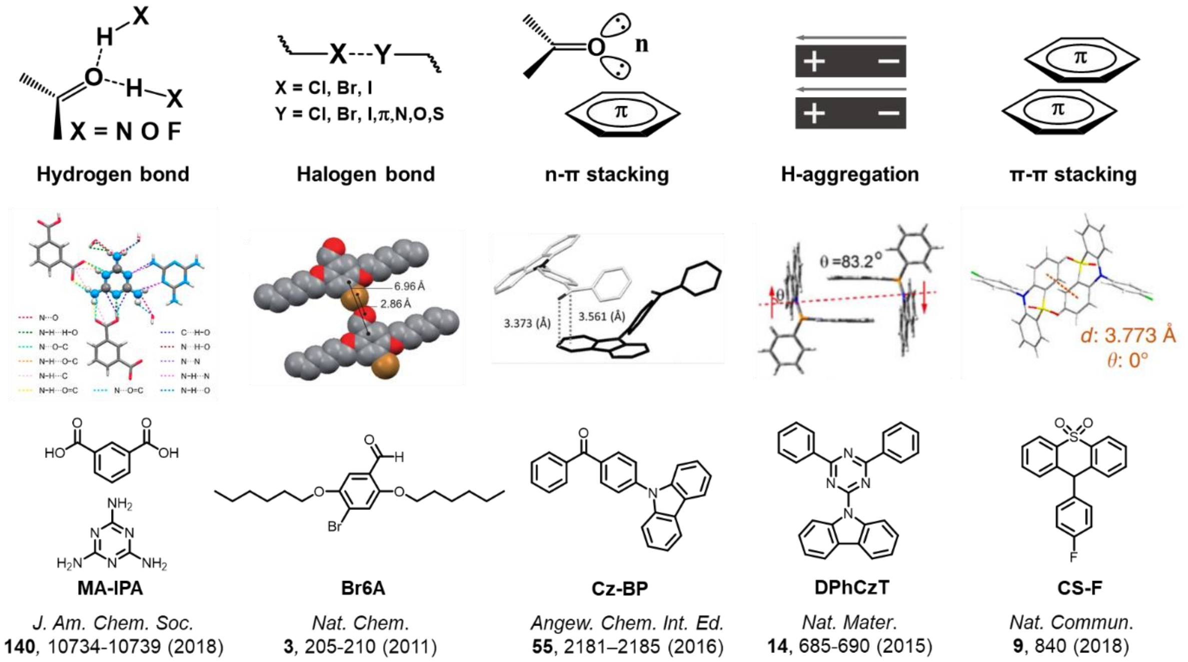

Chart S1 Molecular structures and the packing modes in dimer of the reported persistent RTP materials. **MA-IPA** aggregates containing intermolecular hydrogen bonds achieve an ultralong emission lifetime of up to 1.91 s and a high phosphorescence quantum efficiency of 24.3%. Halogen bonding in **Br6A** crystal can be made to direct the heavy atom effect to produce high phosphorescent quantum yields of up to 2.9%. RTP with lifetime of 0.49 s resuting from intermolecular electronic coupling was obtained in **Cz-BP** crystal. Yellow-green phosphorescence lifetime up to 1.05 s was achieved by effective stabilization of triplet excited states through strong coupling in **DPhCzT** crystal with H-aggregation. Strong π–π interactions in **CS-F** crystal can also promote the RTP lifetime. Copyright 2018 American Chemistry Society, 2011 Nature Publishing Group, 2016 Wiley-VCH, 2015 Nature Publishing Group, 2018 Nature Publishing Group.

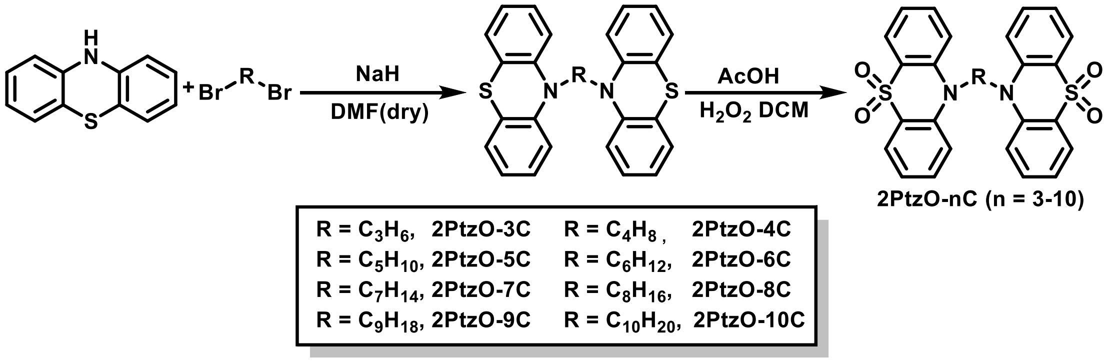

Scheme S1 The synthetic route of **2PtzO-nC**.

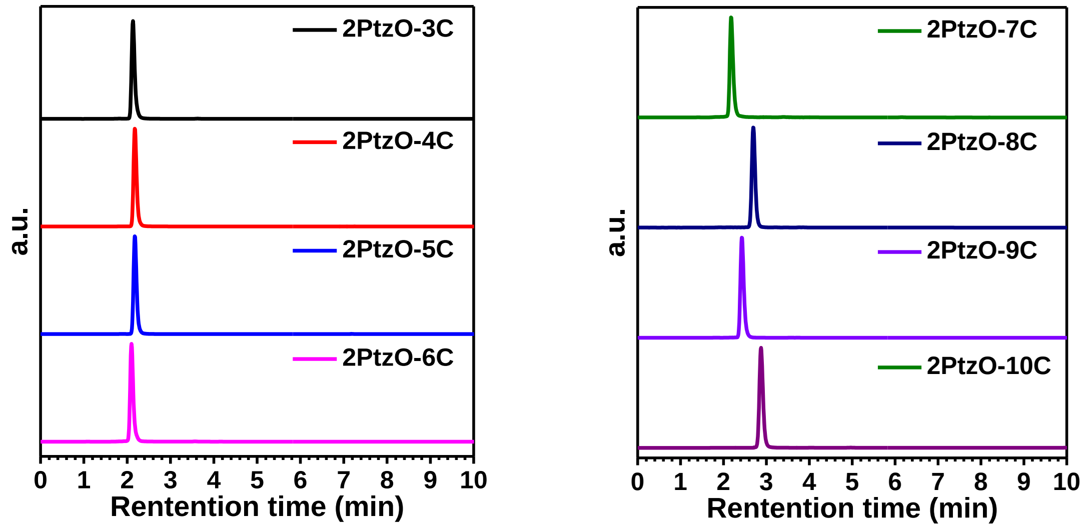

Fig. S1 High performance liquid chromatography diagrams of **2PtzO-nC**.

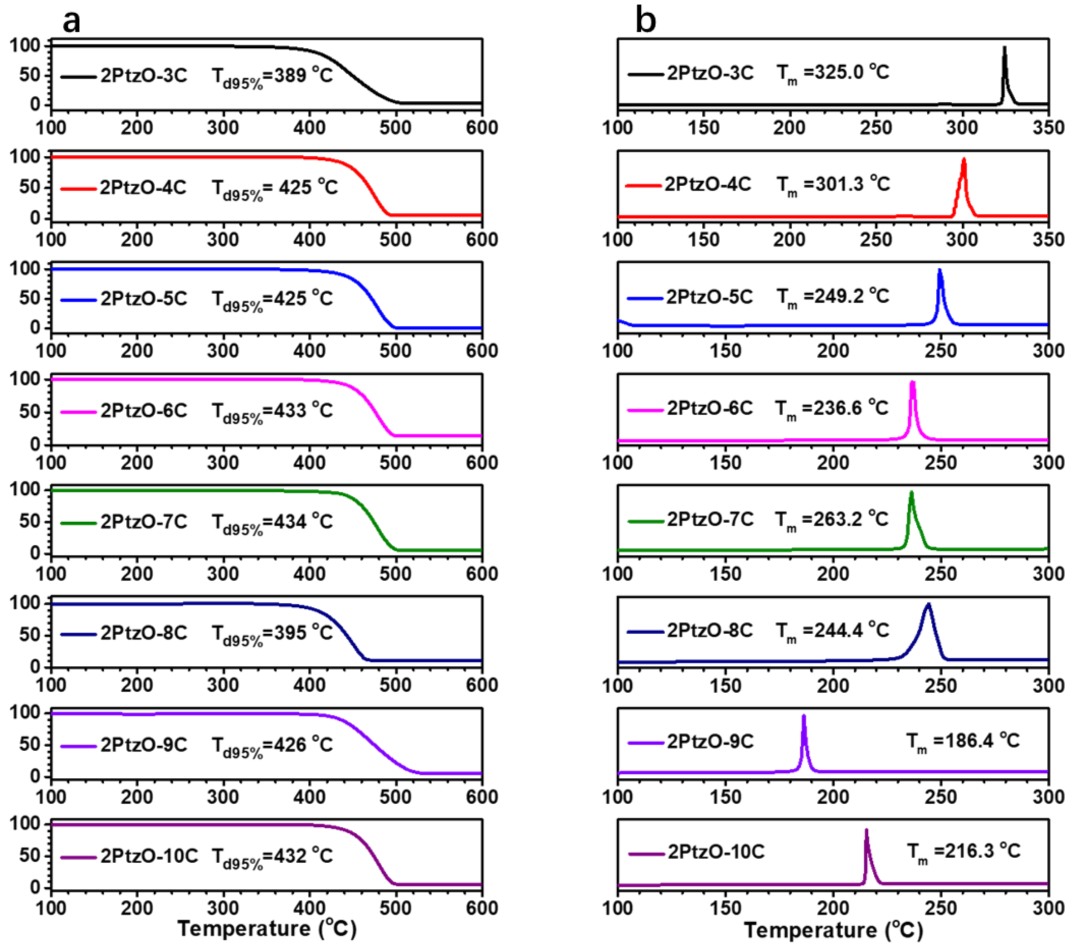

Fig. S2 a Thermo-gravimetric Analysis (TGA) curves. **b** Differential scanning calorimeter (DSC) curves of **2PtzO-nC**.

Table S1 Photophysical data of **2PtzO-nC** (n = 3-6) in solutions and crystals.

**compound**	**2PtzO-3C**	**2PtzO-4C**	**2PtzO-5C**	**2PtzO-6C**
$\lambda_{abs}{}^{a}$ (nm)	334	335	337	337
$\lambda_{abs}{}^{b}$ (nm)	337	337	340	338
$\lambda_{F}{}^{c}$ (nm)	361	362	364	368
$\lambda_{P}{}^{c}$ (nm)	400	409	406	404
$\tau_{P}{}^{c}$ (ms)	376.5	488.3	451.1	383.0
$\lambda_{P}{}^{d}$ (nm)	448, 491	448, 484	471	461
$\tau_{P}{}^{d}$ (ms)	1292.9, 1465.4	605.8, 593.3	1618.2, 672.9	449.6, 765.6
$\lambda_{F}{}^{e}$ (nm)	384	385	380	384
$\tau_{F}{}^{e}$ (ns)	1.06	1.06	1.02	1.04
$\lambda_{P}{}^{e}$ (nm)	493	441, 502	444, 512	445, 503
$\tau_{P}{}^{e}$ (ms)	59.8	119.9, 78.6	107.3, 156.7	142.8, 256.1
$\Phi_{PL}{}^{e}$(%)	6.32	3.96	6.58	3.69
$\lambda_{P}{}^{f}$ (nm)	404	405	405	404
$\tau_{P}{}^{f}$ (ms)	363.0	359.6	364.8	375.0

[a] Observed from absorption spectra in dilute dichloromethane solution.

[b] Observed from absorption spectra in solid powder state.

[c] Determined in dilute dichloromethane solution at 77K.

[d] Determined in crystal state at 77K.

[e] Determined in crystal state at room temperature.

[f] Determined in PMMA film at 77K.

* The left of $\tau_{P}{}^{d}$ is the phosphorescence lifetime at 445 nm and the right is at 500 nm.

Table S2 Photophysical data of **2PtzO-nC** (n = 7-10) in solutions and crystals.

compound	2PtzO-7C	2PtzO-8C	2PtzO-9C	2PtzO-10C
$\lambda_{abs}{}^{a}$ (nm)	338	337	337	337
$\lambda_{abs}{}^{b}$ (nm)	339	338	340	339
$\lambda_{F}{}^{c}$ (nm)	364	367	364	362
$\lambda_{P}{}^{c}$ (nm)	406	406	405	405
$\tau_{P}{}^{c}$ (ms)	320.5	360.0	337.1	351.6
$\lambda_{P}{}^{d}$ (nm)	443, 494	448, 486	456	443, 489
$\tau_{P}{}^{d}$ (ms)	2163.2, 2128.5	314.6, 318.4	1696.9, 1596.5	1435.5, 1239.8
$\lambda_{F}{}^{e}$ (nm)	378	378	376	380
$\tau_{F}{}^{e}$ (ns)	0.88	0.95	0.94	0.93
$\lambda_{P}{}^{e}$ (nm)	501	494	437, 509	500
$\tau_{P}{}^{e}$ (ms)	86.0	60.4	30.3, 125.7	90.8
$\Phi_{PL}{}^{e}$ (%)	12.80	10.44	1.12	3.13
$\lambda_{P}{}^{f}$ (nm)	406	405	404	404
$\tau_{P}{}^{f}$ (ms)	358.1	343.1	349.6	358.4

[a] Observed from absorption spectra in dilute dichloromethane solution.

[b] Observed from absorption spectra in solid powder state.

[c] Determined in dilute dichloromethane solution at 77K.

[d] Determined in crystal state at 77K.

[e] Determined in crystal state at room temperature.

* The left of $\tau_{P}{}^{d}$ is the phosphorescence lifetime at 445 nm and the right is at 500 nm.

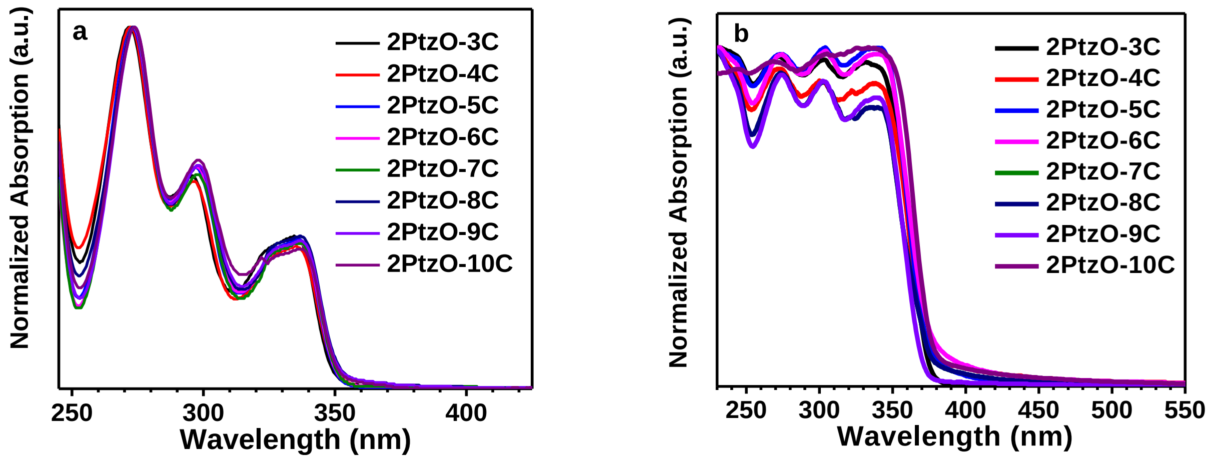

Fig. S3 a The UV-vis absorption spectra of **2PtzO-nC** in the dilute DCM solution (10 μM) and (**b**) in solid state.

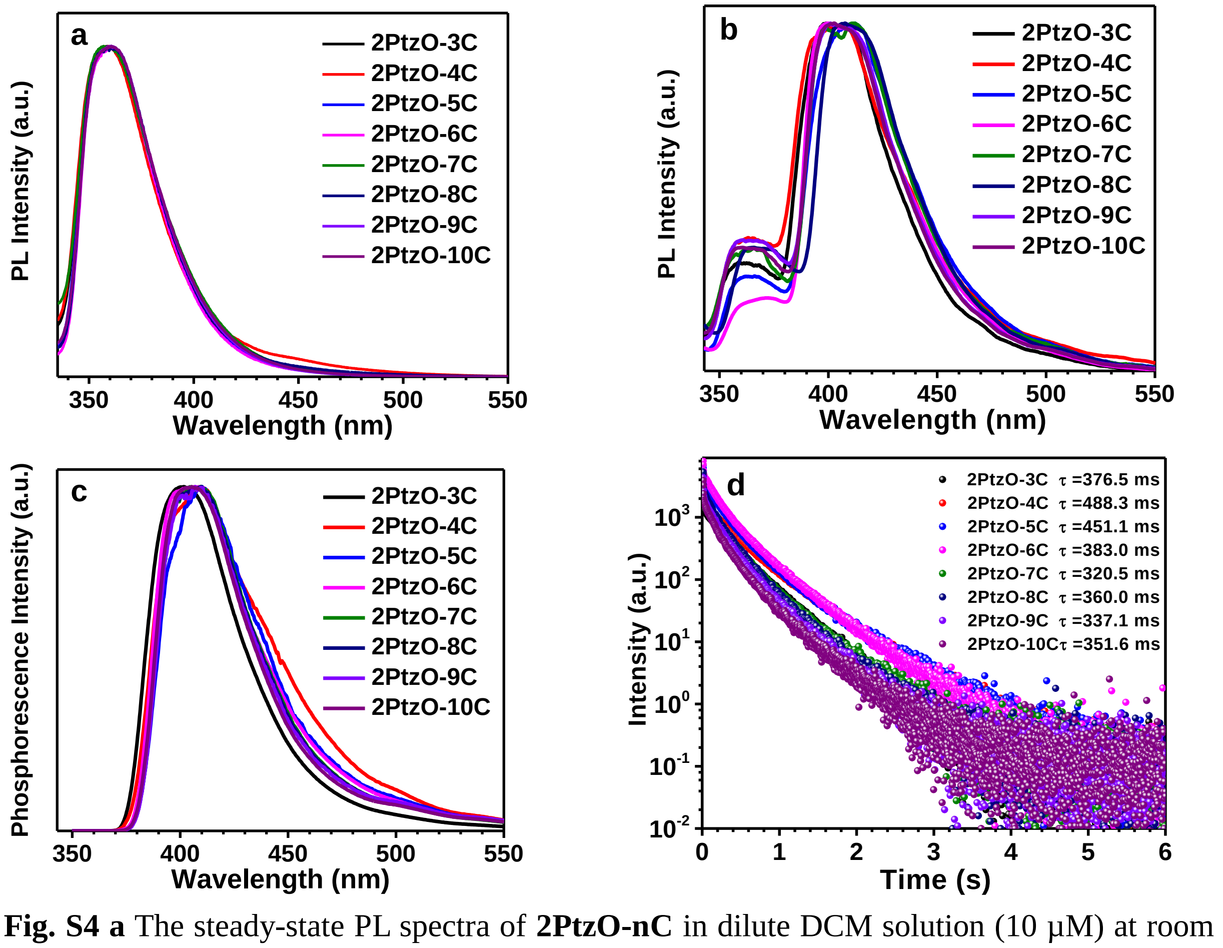

Fig. S4 a The steady-state PL spectra of **2PtzO-nC** in dilute DCM solution (10 μM) at room temperature and **b**) at 77K (λ_{ex} = 330 nm). **c** The phosphorescence spectra of **2PtzO-nC** in dilute DCM solution (10 μM) at 77K (λ_{ex} = 330 nm). **d** The phosphorescence-decay curves of **2PtzO-nC** in dilute DCM solution (10 μM) at 77K (@ 405 nm; λ_{ex} = 330 nm).

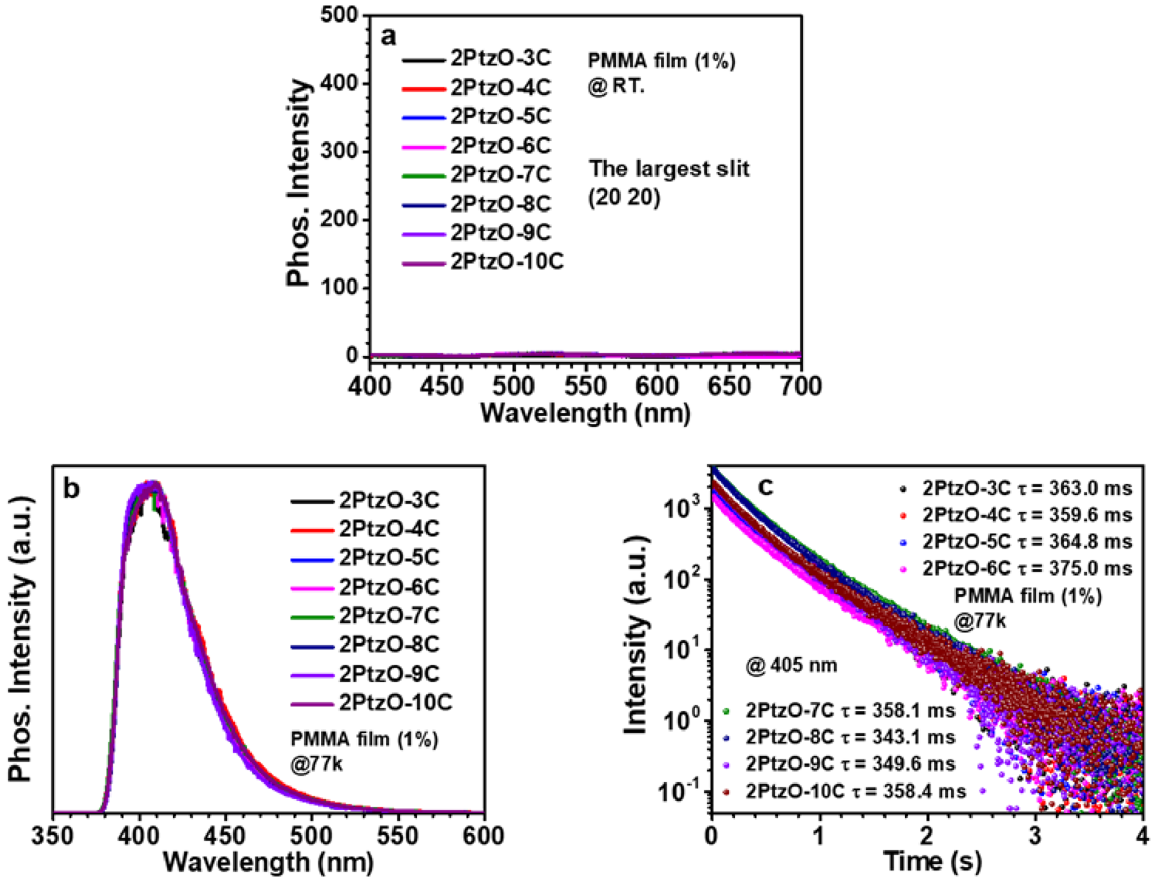

Fig. S5 **a** The phosphorescence spectra of **2PtzO-nC** doped in polymethyl methacrylate (PMMA) matrix with mass ratio of 1% at room temperture (λ_{ex} = 365 nm); **b** The phosphorescence spectra of **2PtzO-nC** doped in polymethyl methacrylate (PMMA) matrix with mass ratio of 1% at 77K (λ_{ex} = 330 nm). **c** Time-resolved phosphorescence-decay curves of **2PtzO-nC** in polymethyl methacrylate (PMMA) matrix at 77K (@ 405 nm; λ_{ex} = 330 nm).

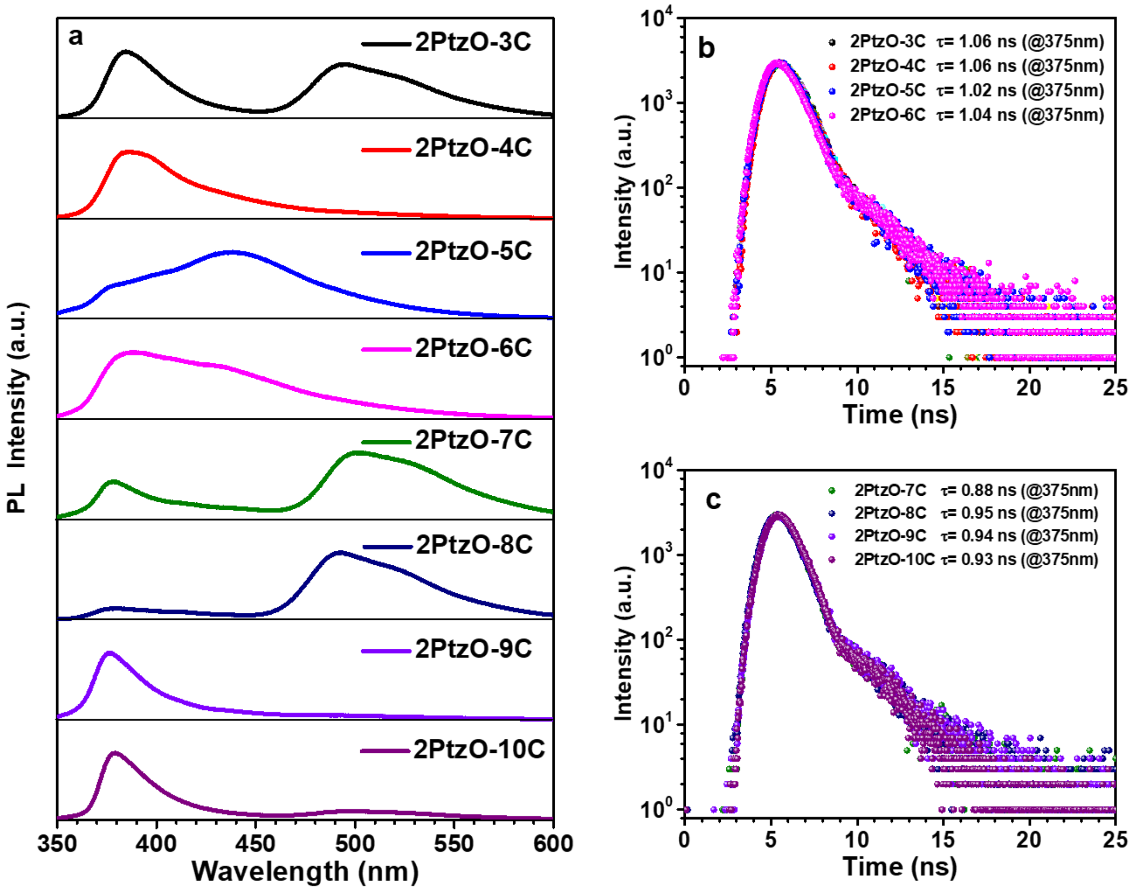

Fig. S6 a The steady-state PL spectra of **2PtzO-nC** in crystal state at room temperature (λ_{ex} = 330 nm). **b** PL decay curves of **2PtzO-nC** (n = 3-6) in crystal state (λ_{ex} = 330 nm; monomer fluorescence). **c** PL decay curves of **2PtzO-nC** (n = 7-10) in crystal state (λ_{ex} = 330 nm; monomer fluorescence).

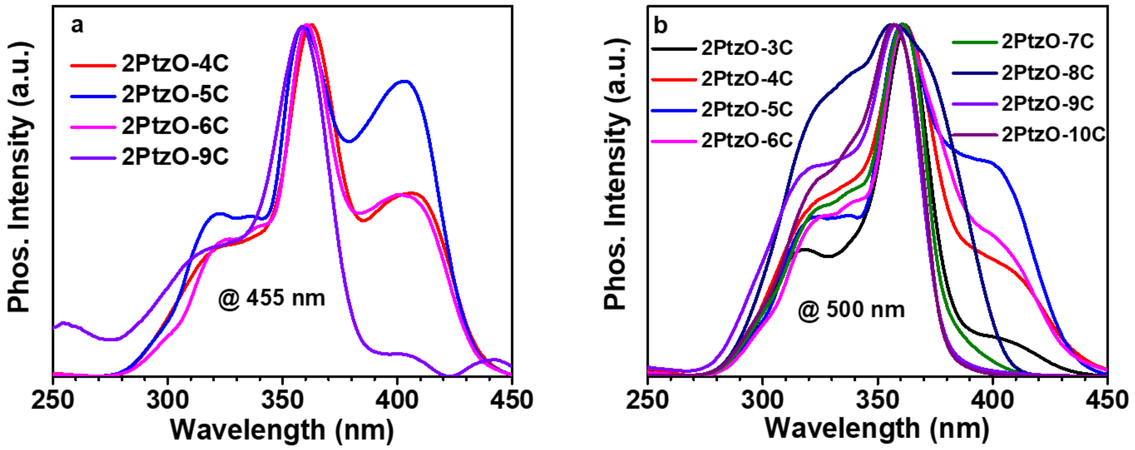

Fig. S7 a The excitation spectra of **2PtzO-nC** in crystal state for the phosphorescence band at 445 nm and **b** at 500 nm.

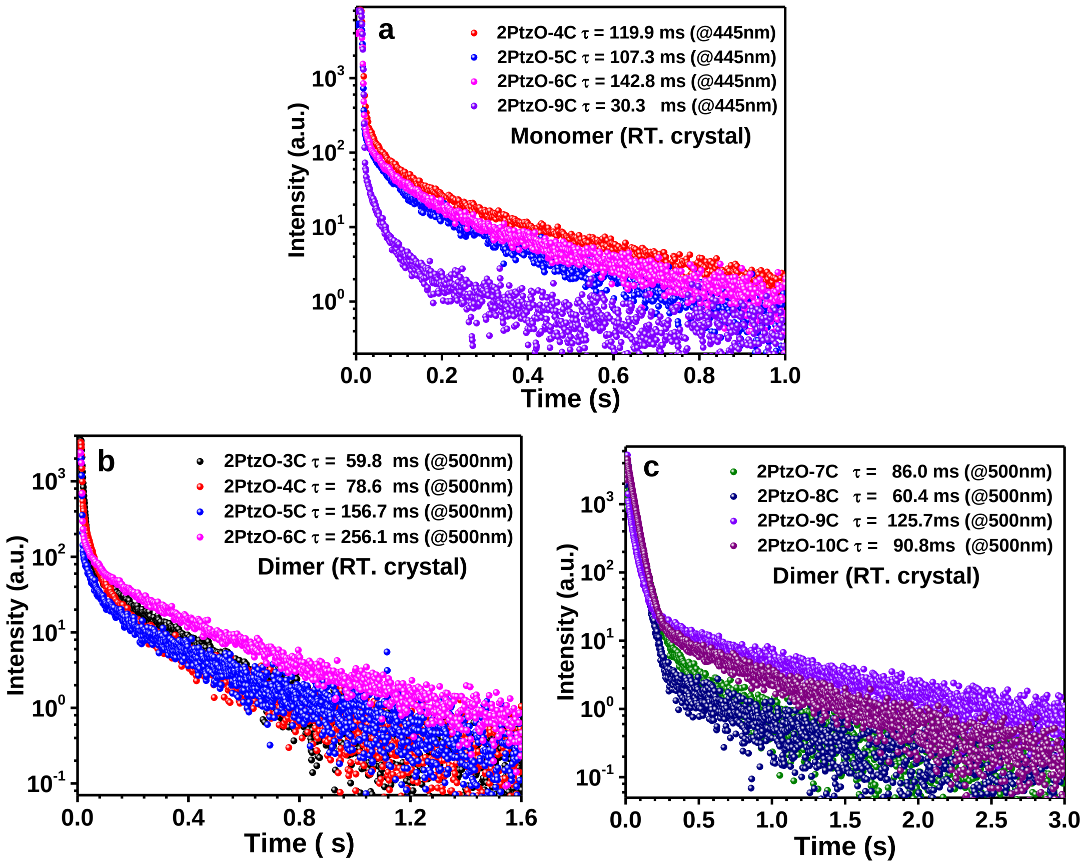

Fig. S8 a Time-resolved RTP-decay curves of **2PtzO-nC** crystals at 445 nm (λ_{ex} = 365 nm; monomer phosphorescence). **b** Time-resolved RTP-decay curves of **2PtzO-nC** (n = 3-6) crystals at 500 nm (λ_{ex} = 365 nm; dimer phosphorescence). **c** Time-resolved RTP-decay curves of **2PtzO-nC** (n = 7-10) crystals at 500 nm (λ_{ex} = 365 nm; dimer phosphorescence).

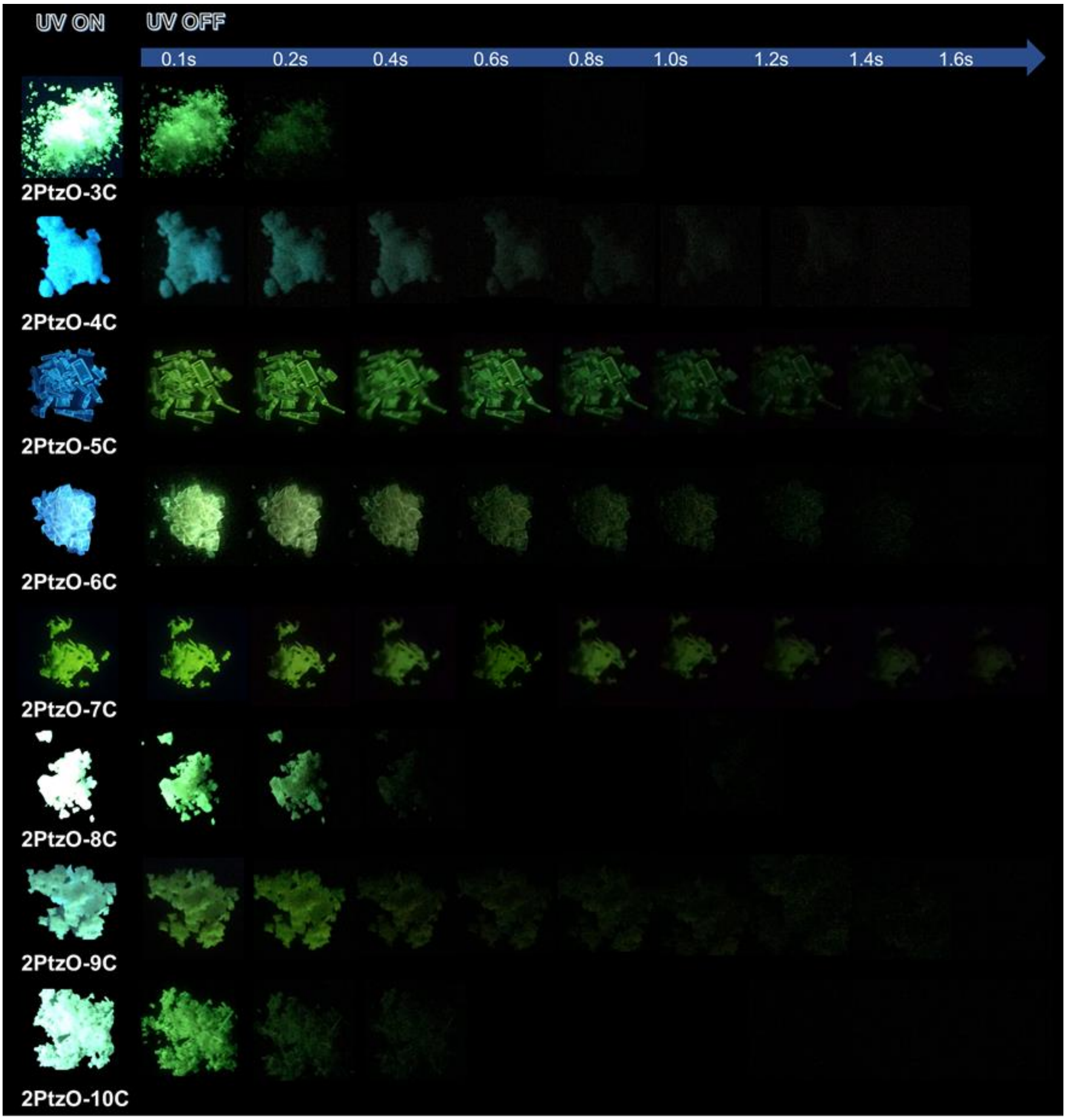

Fig. S9 Photographs of **2PtzO-nC** crystals taken at different times before and after removing the excitation of a 365 nm UV lamp.

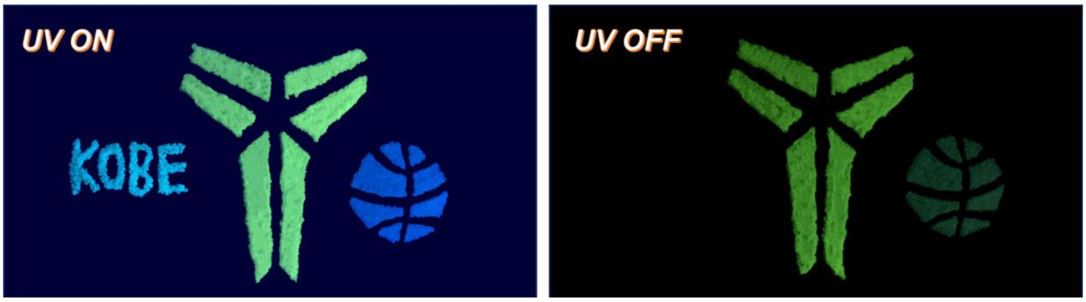

Fig. S10 Application of multiple anti-counterfeiting: the name of "KOBE" was written by pyrene—a conventional dimer fluorescent material; "Mamba" logo was written by **2PtzO-7C** powder—pure dimer phosphorescence material and "basketball" written by **2PtzO-6C** powder—dual phosphorescence emissions material (left: under 365 nm UV lamp; right: after the removal of 365 nm UV excitation). A very meaningful moral: Kobe is dead, but Mamba Mentality and basketball faith are forever.

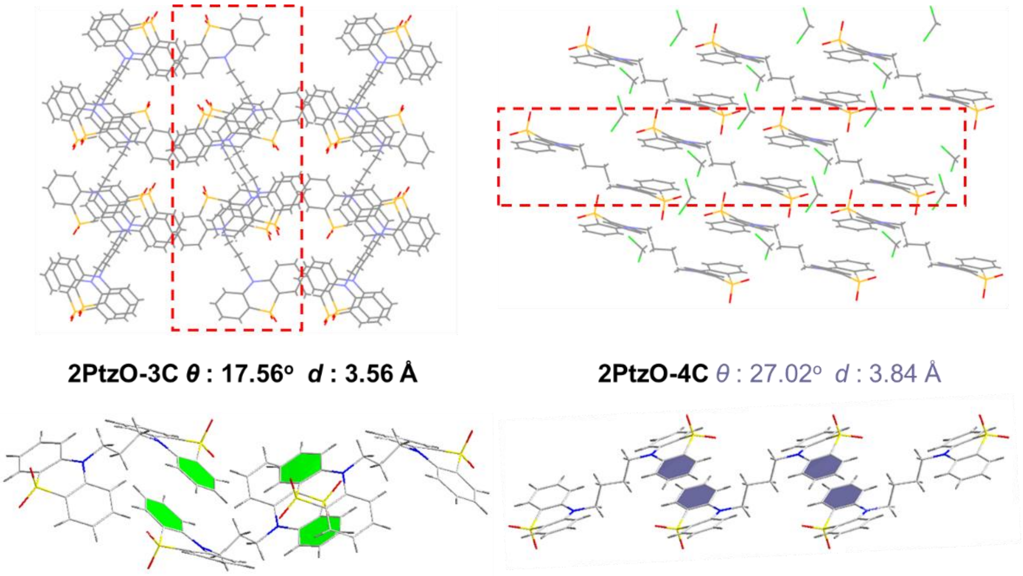

Fig. S11 Entire and local packing modes of **2PtzO-3C** and **2PtzO-4C**. Phenyl rings involved in the stronger π–π interaction is labeled by green color and weaker π–π interaction by indigo blue color. (*d*: vertical distance between the adjacent benzene planes; *θ*: displacement angle, the angle between the centroid–centroid line and the vertical line)

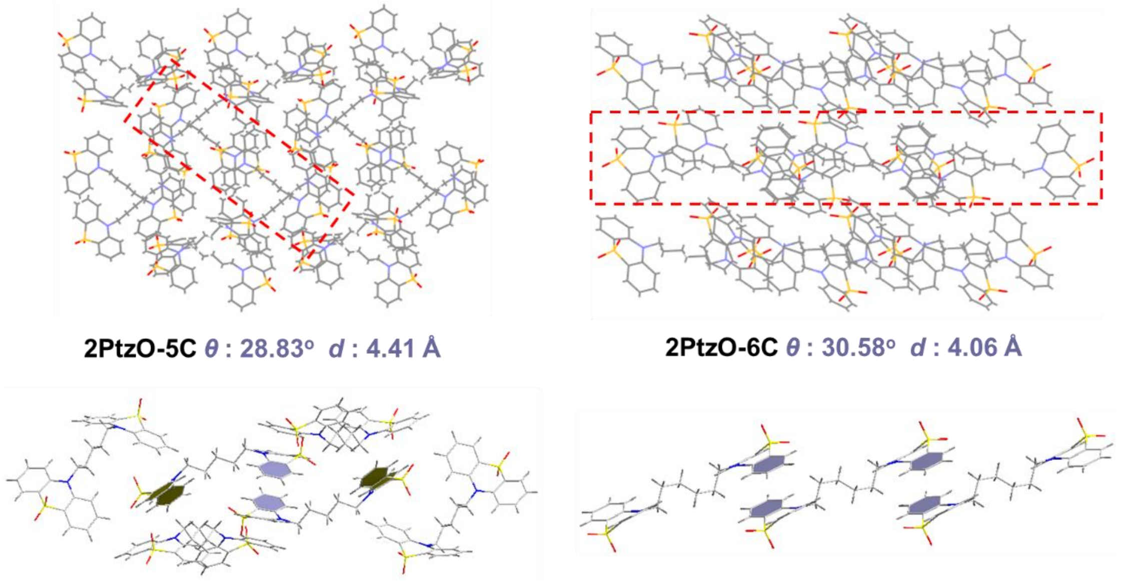

Fig. S12 Entire and local packing modes of **2PtzO-5C** and **2PtzO-6C**. Phenyl rings involved in weak π–π interaction is labeled by indigo blue color. (*d*: vertical distance between the adjacent benzene planes; *θ*: displacement angle, the angle between the centroid–centroid line and the vertical line)

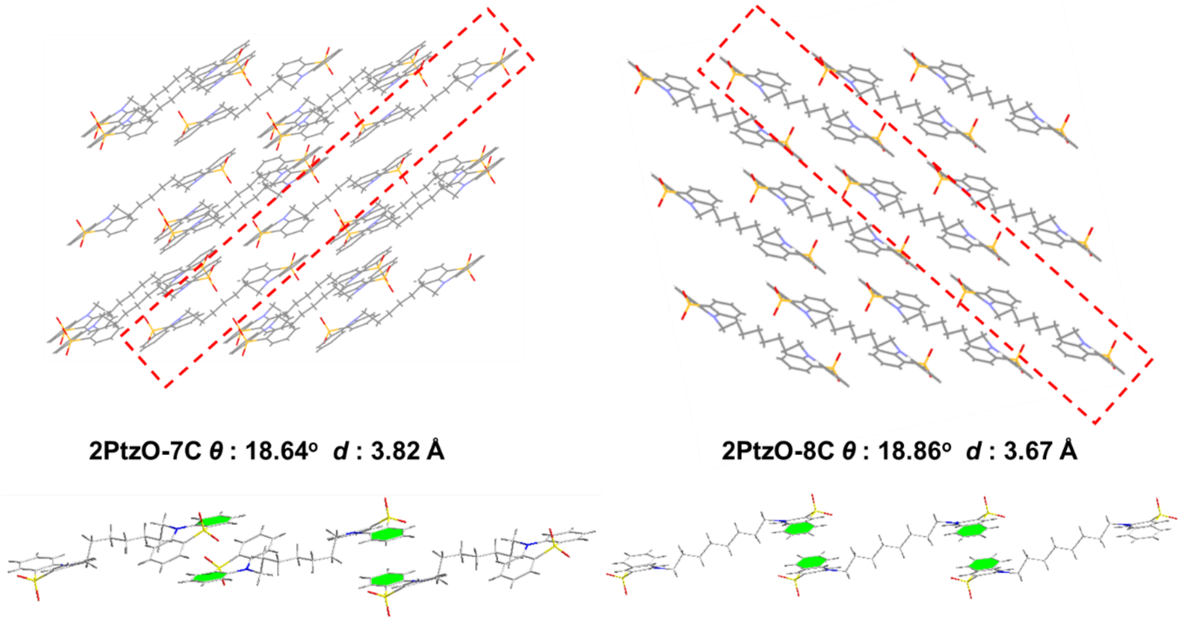

Fig. S13 Entire and local packing modes of **2PtzO-7C** and **2PtzO-8C**. Phenyl rings involved in the strong π–π interaction is labeled by green color. (*d*: vertical distance between the adjacent benzene planes; *θ*: displacement angle, the angle between the centroid–centroid line and the vertical line)

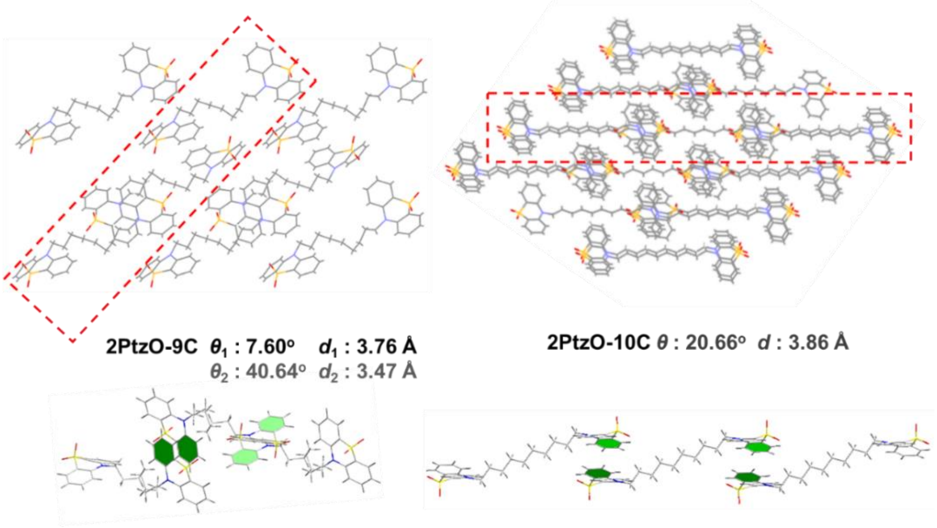

Fig. S14 Entire and local packing modes of **2PtzO-9C** and **2PtzO-10C**. Phenyl rings involved in the strong π–π interaction is labeled by green color. (*d*: vertical distance between the adjacent benzene planes; *θ*: displacement angle, the angle between the centroid–centroid line and the vertical line)

Table S3 Structure data of single crystals of **2PtzO-nC** (n = 3-6).

Name	**2PtzO-3C**	**2PtzO-4C**	**2PtzO-5C**	**2PtzO-6C**
Formula	$C_{27}H_{22}N_2O_4S_2$	$C_{28}H_{24}N_2O_4S_2$	$C_{29}H_{26}N_2O_4S_2$	$C_{30}H_{28}N_2O_4S_2$
Wavelength(Å)	1.54184	1.54184	1.54184	1.54184
Space Group	P b c n	C c	P b c a	P 21/c
Cell Lengths (Å)	a=20.2922(2)	a=9.6965(3)	a=16.3020(10)	a=11.6438(2)
	b=7.95270(10)	b=13.3896(4)	b=11.8701(10)	b=15.2057(2)
	c=14.2127(10)	c=21.1235(6)	c=26.1081(10)	c=14.6760(3)
Cell Angles (º)	α=90	α=90	α=90	α=90
	β=90	β=101.429(3)	β=90	β=90.012(2)
	γ=90	γ=90	γ=90	γ=90
Cell Volume (Å^3)	2293.61(4)	2688.12(14)	5052.08(6)	2598.42(8)
Z	4	4	8	4
Density (g/cm^3)	1.456	1.486	1.395	1.392
F (000)	1048	1248	2224	1144
h_{max}, k_{max}, l_{max}	24,9,16	11,15,25	20,14,32	13,18,17
CCDC Number	2088011	2088093	2088010	2088094

Table S4 Structure data of single crystals of **2PtzO-nC** (n = 7-10).

Name	**2PtzO-7C**	**2PtzO-8C**	**2PtzO-9C**	**2PtzO-10C**
Formula	$C_{31}H_{30}N_2O_4S_2$	$C_{32}H_{32}N_2O_4S_2$	$C_{33}H_{34}N_2O_4S_2$	$C_{34}H_{36}N_2O_4S_2$
Wavelength(Å)	1.54184	1.54184	1.54184	1.54184
Space Group	C 2/c	P -1	P -1	I 2/a
Cell Lengths (Å)	a=18.5127(2)	a=7.7745(5)	a=8.6717(2)	a=16.6374(5)
	b=9.29390(10)	b=9.2795(5)	b=12.3273(6)	b=10.0062(3)
	c=15.3722(2)	c=10.3305(7)	c=13.6657(7)	c=19.6938(6)
Cell Angles (°)	α=90	α=110.901(6)	α=90.840(4)	α=90
	β=98.7040(10)	β=95.509(6)	β=103.087(3)	β=109.222(4)
	γ=90	γ=98.111(5)	γ=92.202(3)	γ=90
Cell Volume ($Å^3$)	2614.41(5)	680.59(8)	1421.44(11)	3095.79(18)
Z	4	1	2	4
Density (g/cm^3)	1.419	1.397	1.371	1.289
F (000)	1176	302.0	620.0	1272
h_{max}, k_{max}, l_{max}	22,11,18	9,11,12	10,15,16	19,11,23
CCDC Number	2088095	2088096	2088097	2088098

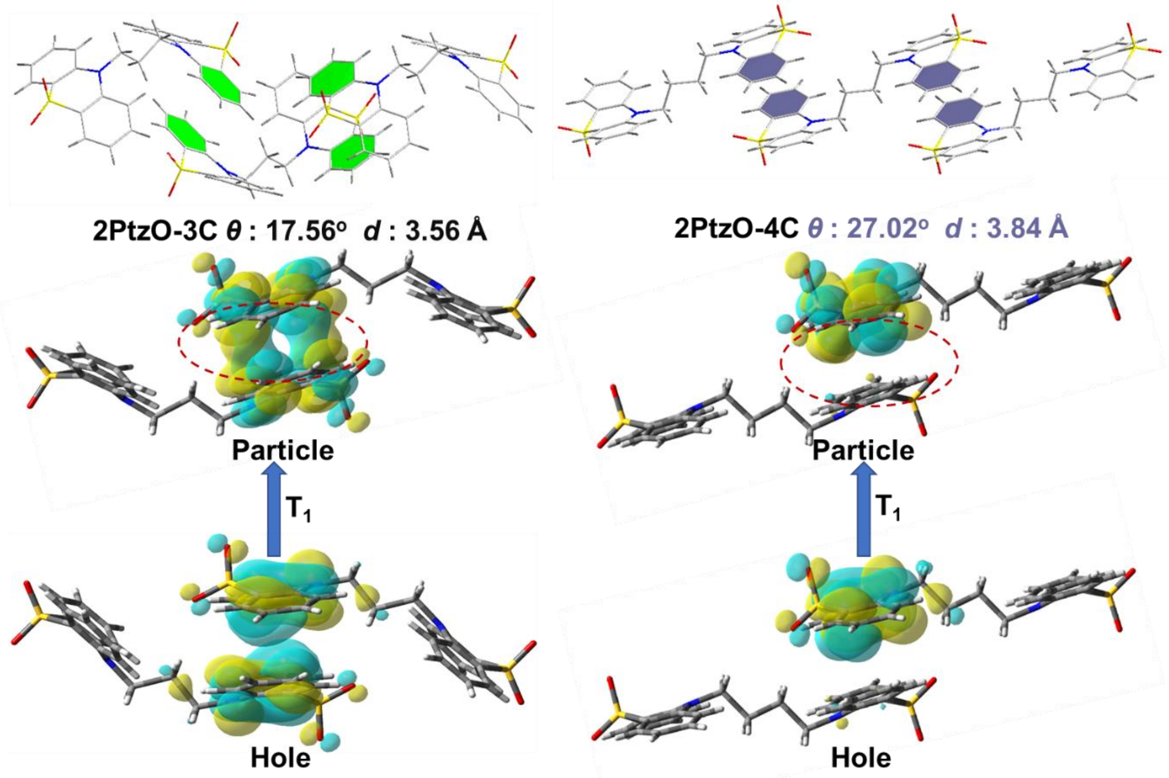

Fig. S15 Natural transition orbitals (NTOs) of the T_1 state for the dimers of **2PtzO-3C** and **2PtzO-4C**. (The tighter the π-π stacking, the stronger the orbital coupling in T_1 state).

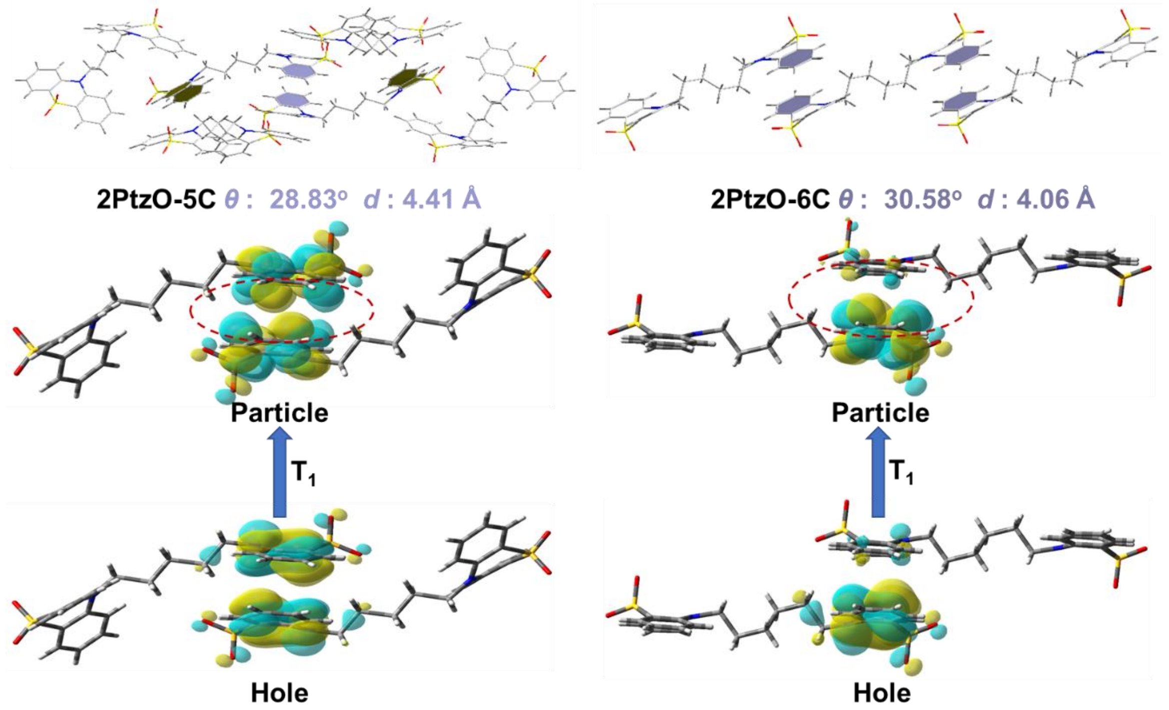

Fig. S16 Natural transition orbitals (NTOs) of T_1 state for the dimers of **2PtzO-5C** and **2PtzO-6C**. (The tighter the π-π stacking, the stronger the orbital coupling in T_1 state).

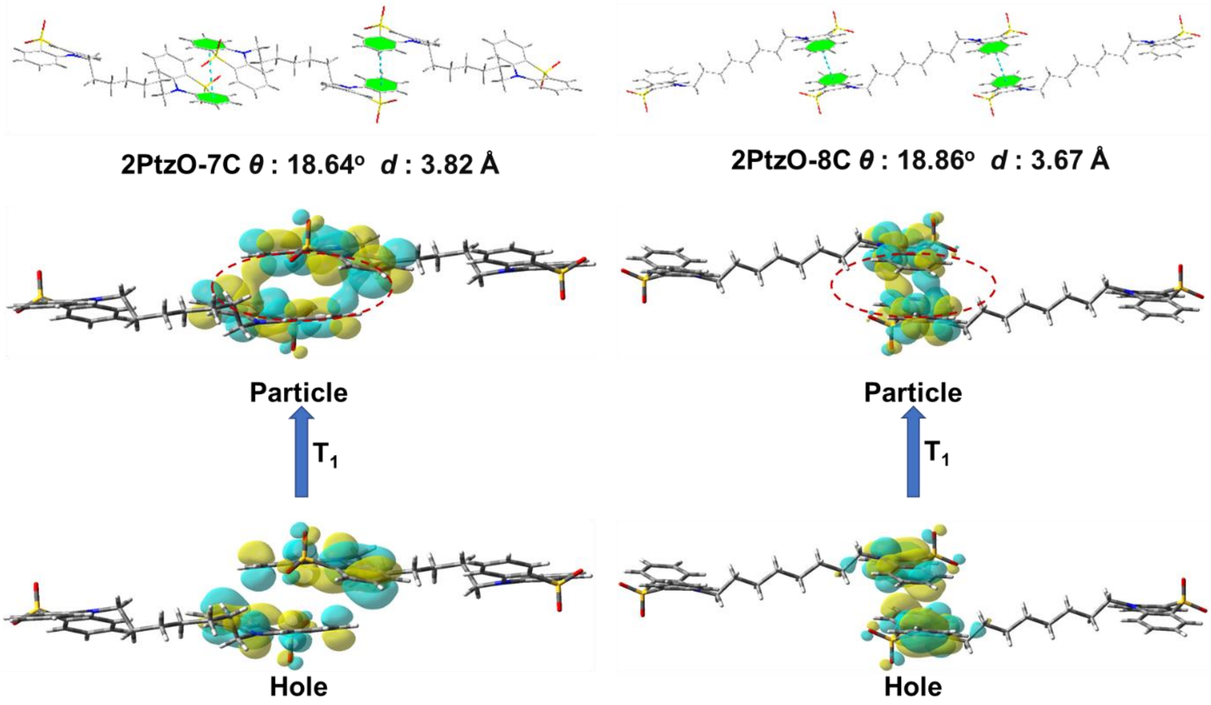

Fig. S17 Natural transition orbitals (NTOs) of T_1 state for the dimers of **2PtzO-7C** and **2PtzO-8C**. (The tighter the π-π stacking, the stronger the orbital coupling in T_1 state).

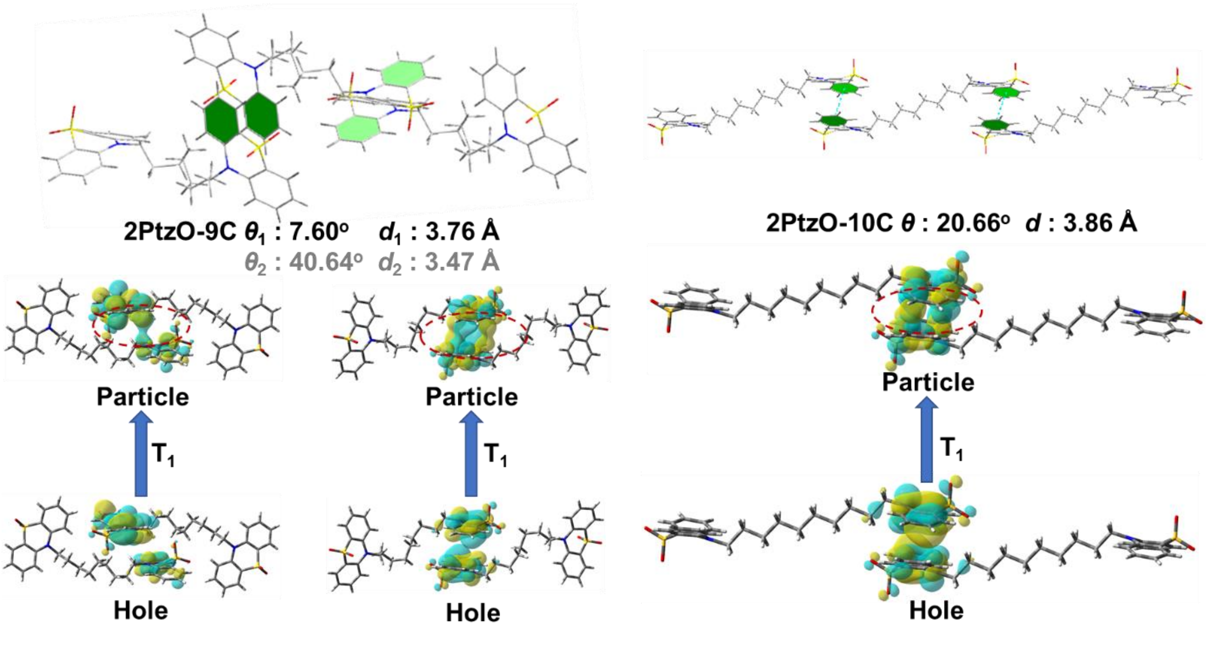

Fig. S18 Natural transition orbitals (NTOs) of T_1 state for the dimers of **2PtzO-9C** and **2PtzO-10C**. (The tighter the π-π stacking, the stronger the orbital coupling in T_1 state).

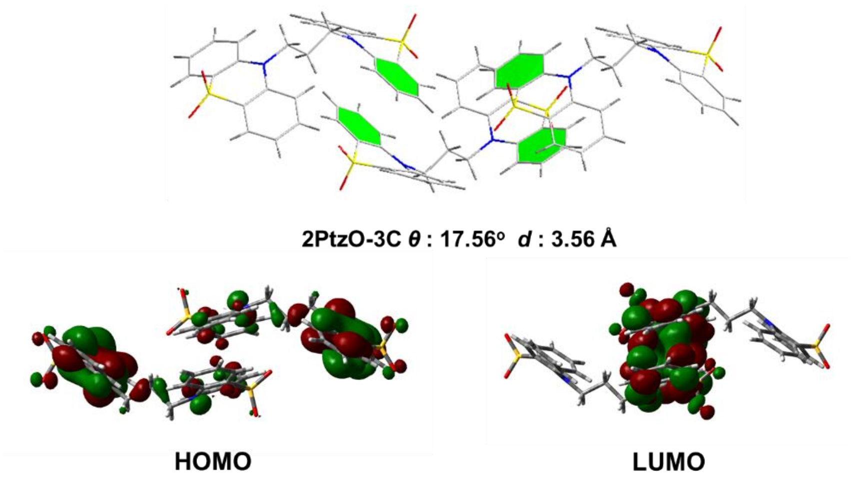

Fig. S19 The HOMO, LUMO orbital distributions for the dimer unit of **2PtzO-3C**.

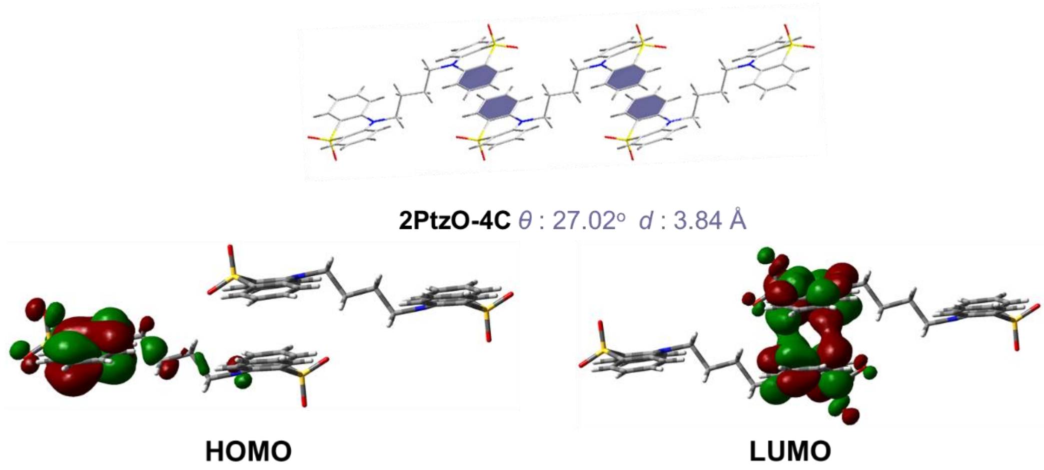

Fig. S20 The HOMO, LUMO orbital distributions for the dimer unit of **2PtzO-4C**.

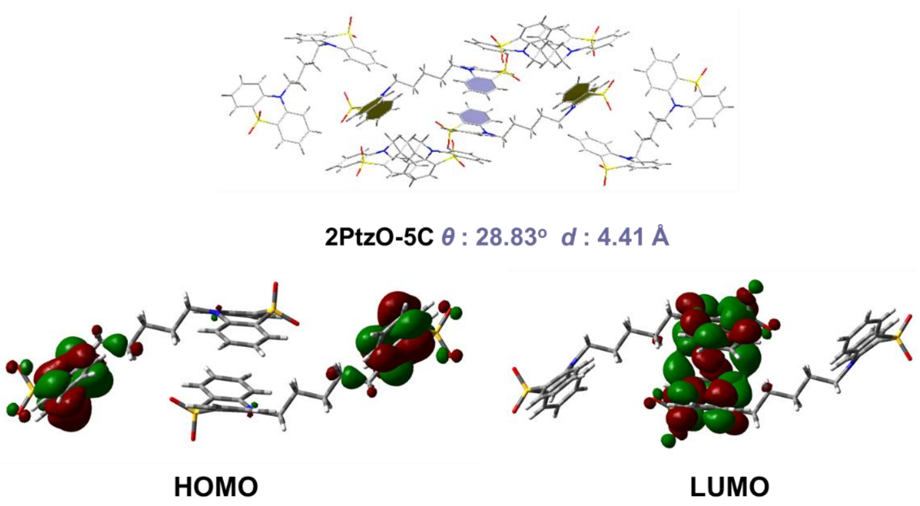

Fig. S21 The HOMO, LUMO orbital distributions for the dimer unit of **2PtzO-5C**.

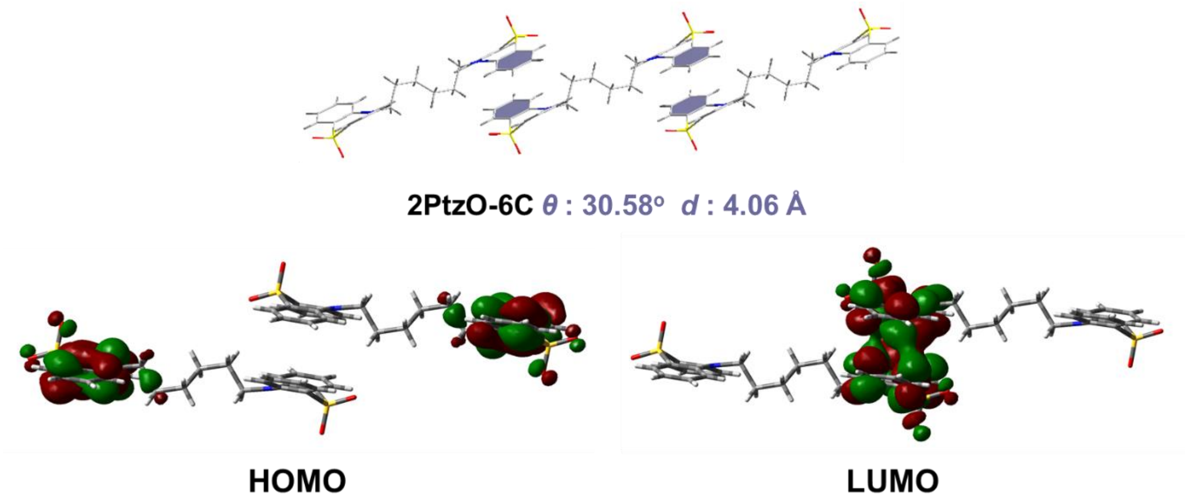

Fig. S22 The HOMO, LUMO orbital distributions for the dimer unit of **2PtzO-6C**.

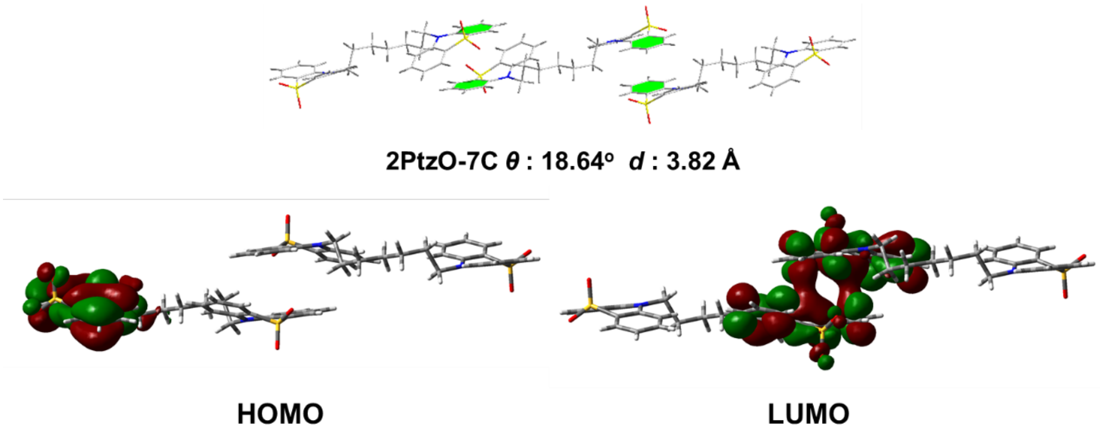

Fig. S23 The HOMO, LUMO orbital distributions for the dimer unit of **2PtzO-7C**.

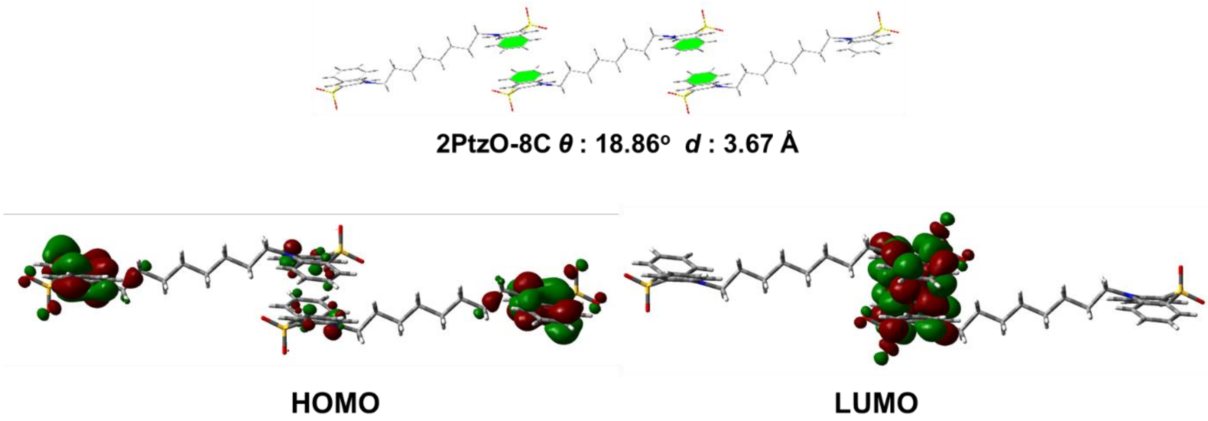

Fig. S24 The HOMO, LUMO orbital distributions for the dimer unit of **2PtzO-8C**.

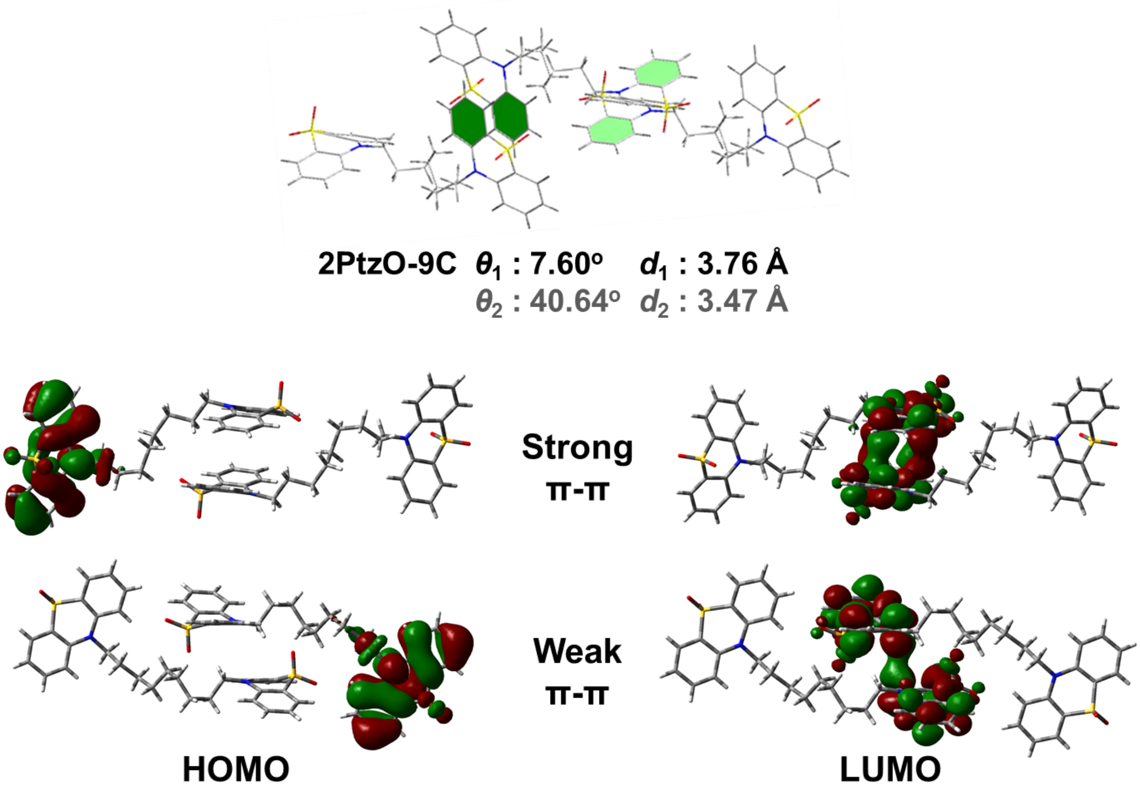

Fig. S25 The HOMO, LUMO orbital distributions for the dimer units of **2PtzO-9C**.

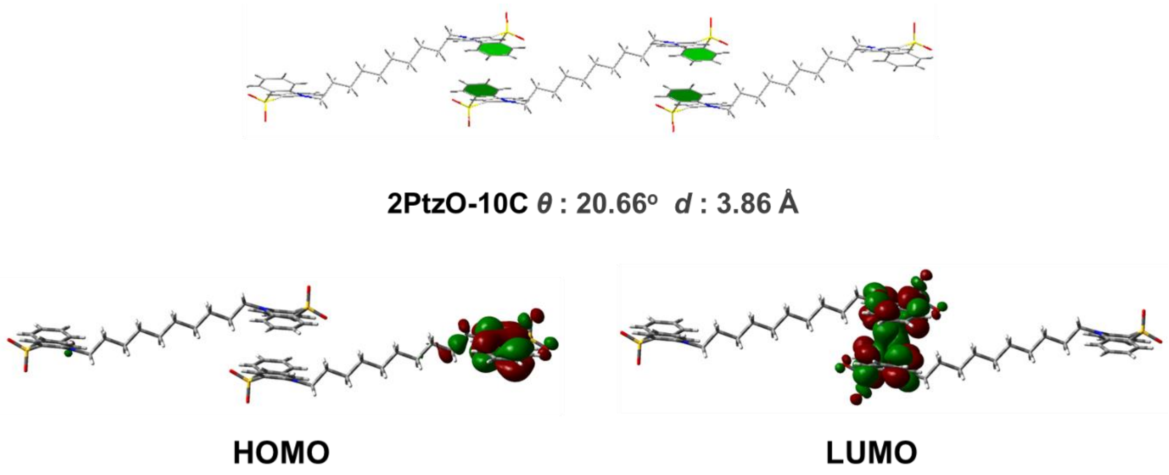

Fig. S26 The HOMO, LUMO orbital distributions for the dimer unit of **2PtzO-10C**.

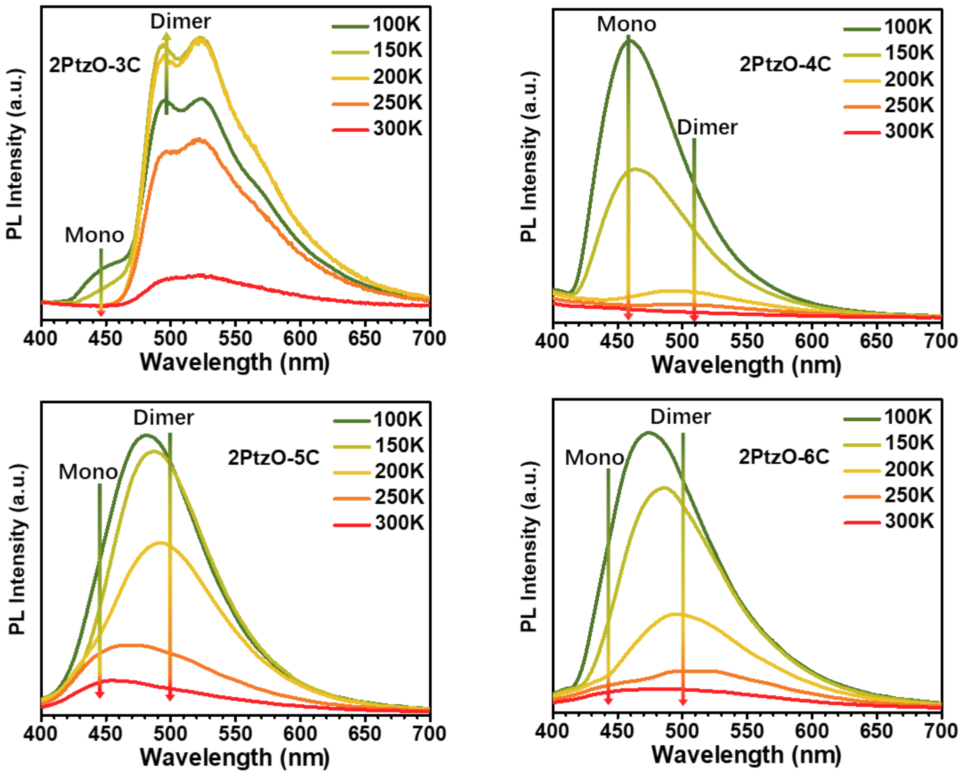

Fig. S27 The PL spectra of **2PtzO-nC** (n = 3-6) crystals at different temperatures.

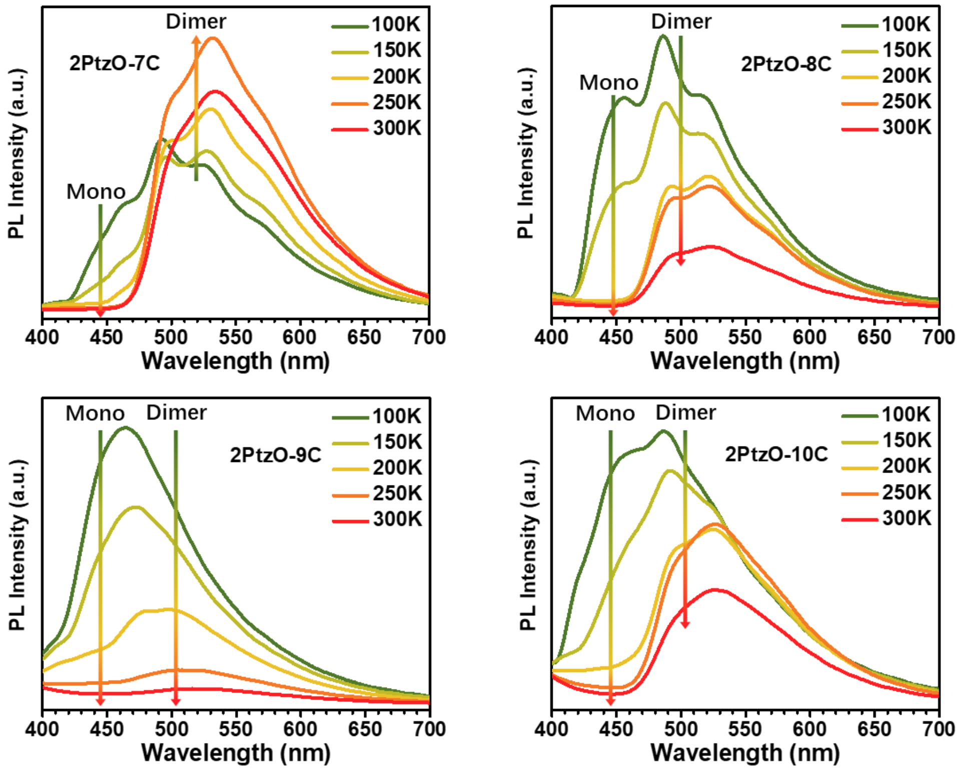

Fig. S28 The PL spectra of **2PtzO-nC** (n = 7-10) crystals at different temperatures.

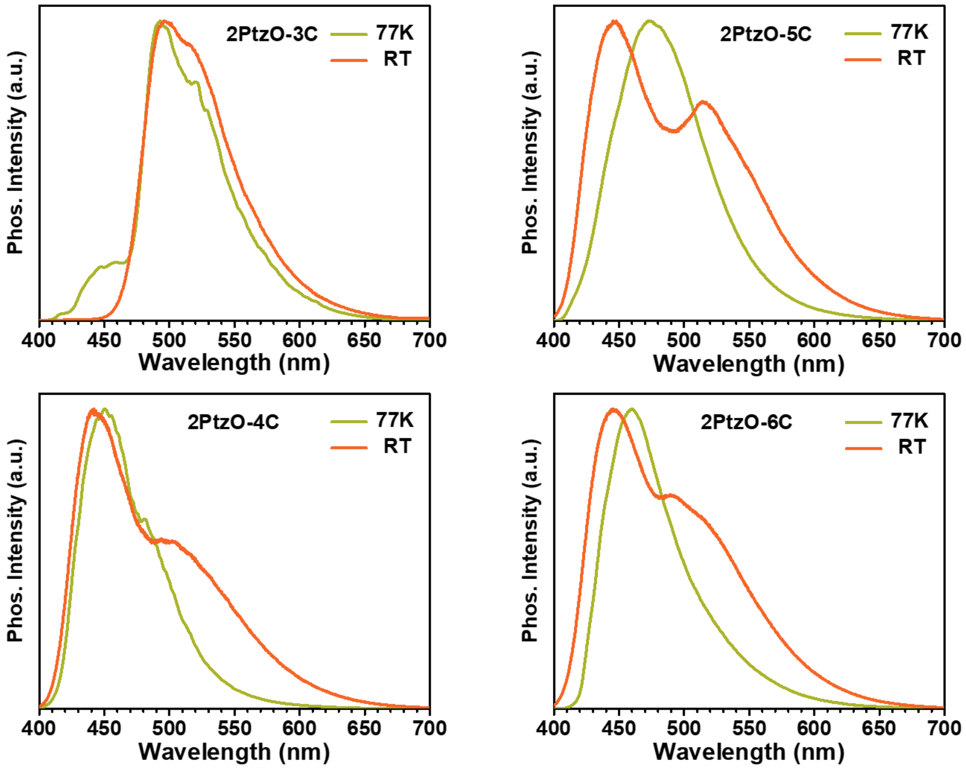

Fig. S29 The normalized phosphorescence spectra of **2PtzO-nC** (n = 3-6) crystals at room temperature and 77 K.

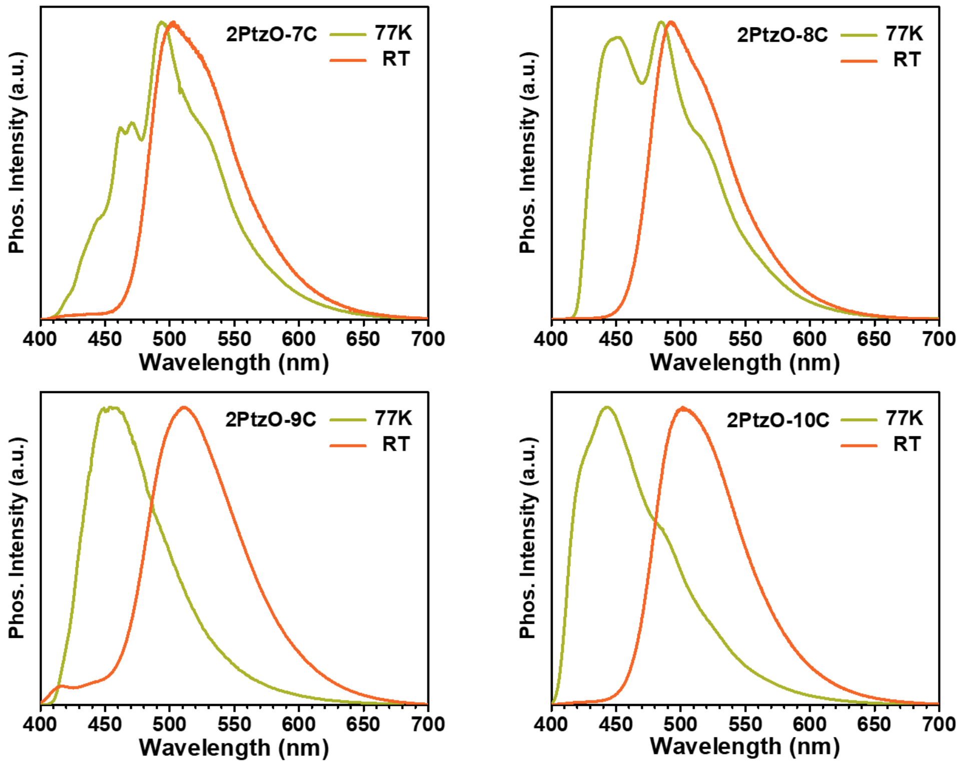

Fig. S30 The normalized phosphorescence spectra of **2PtzO-nC** (n = 7-10) crystals at room temperature and 77 K.

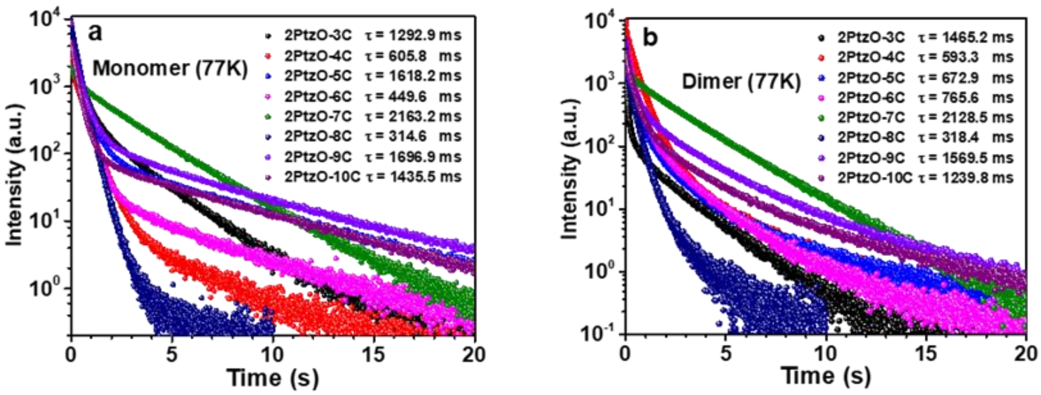

Fig. S31 a Time-resolved PL-decay curves for monomer phosphorescence (@445 nm) of **2PtzO-nC** crystals at 77 K. **b** Time-resolved PL-decay curves for dimer phosphorescence (@500 nm) of **2PtzO-nC** crystals at 77 K.

Table S5 The phosphorescence intensity and ratio of dimer to monomer for **2PtzO-nC** (n = 3-6) crystals at 77 K and room temperature.

	**2PtzO-3C**		**2PtzO-4C**		**2PtzO-5C**		**2PtzO-6C**	
	77 K	RT	77 K	RT	77 K	RT	77 K	RT
I_M **(a.u.)**	0.176	0.007	1.000	1.000	0.739	1.000	0.824	1.000
I_D **(a.u.)**	1.000	1.000	0.302	0.537	0.438	0.714	0.402	0.683
I_D / I_M	5.44	141.41	0.30	0.54	0.59	0.71	0.49	0.68

I_M = Phosphorescence intensity of monomer at about 445 nm in normalized phosphorescence spectra
I_D = Phosphorescence intensity of dimer at about 500 nm in normalized phosphorescence spectra

Table S6 The phosphorescence intensity and ratio of dimer to monomer for **2PtzO-nC** (n = 7-10) crystals at 77 K and room temperature.

	**2PtzO-7C**		**2PtzO-8C**		**2PtzO-9C**		**2PtzO-10C**	
	77 K	RT	77 K	RT	77 K	RT	77 K	RT
I_M **(a.u.)**	0.345	0.019	0.940	0.003	1.000	0.082	1.000	0.023
I_D **(a.u.)**	1.000	1.000	1.000	1.000	0.384	1.000	0.762	1.000
I_D / I_M	2.90	52.63	1.06	333.34	0.38	12.20	0.76	43.48

I_M = Phosphorescence intensity of monomer at about 445 nm in normalized phosphorescence spectra
I_D = Phosphorescence intensity of dimer at about 500 nm in normalized phosphorescence spectra

Notes: The CCDC Numbers for **2PtzO-nC** (n = 3-10) crystals are 2088011, 2088093, 2088010, 2088094, 2088095, 2088096, 2088097 and 2088098 respectively, and the Cif files for these crystals could be downloaded on the website of The Cambridge Crystallographic Data Centre (CCDC) (https://ccdc.cam.ac.uk/).
