# Supplementary material for: Direct demonstration of triplet excimer in purely organic room temperature phosphorescence through rational molecular design
Source: Light Sci Appl. 2022 May 17;11:142. doi: 10.1038/s41377-022-00826-4 (PMC9114335; doi:10.1038/s41377-022-00826-4)
Supplement: Supplementary file 2 — Article confidentiality and copyright transfer agreement [file 41377_2022_826_MOESM2_ESM.pdf]

文章保密与版权转让证明

承　诺　书

此文章不涉密且不存在造假、抄袭、一稿多投等学术不端行为，特此承诺。

第一（通讯）作者签字：刘振江

2022年4月19日

《Light: Science & Applications》编辑部：

我单位 刘振江，田瑜，杨杰，李爱森，王雲生，任佳，方曼曼，唐本忠，李振 作者（需按正式发表文章署名顺序，填写全部作者姓名）为你刊撰写的文章（题目： Direct demonstration of triplet excimer in purely organic room temperature phosphorescence through rational molecular design ），经审查，未发现该文章存在涉密内容和造假、抄袭、一稿多投等学术不端现象。该文章若存在涉密内容和造假、抄袭、一稿多投等学术不端问题，《Light: Science & Applications》编辑部无需承担任何责任。该文章一经录用，其数字化复制权、发行权、汇编权及信息网络传播权将转让予《Light: Science & Applications》编辑部。

导师（课题负责人）签字 李振

单位盖章 天津大学 分子聚集态科学研究院

2022年4月19日
