# Supplementary material for: Direct demonstration of triplet excimer in purely organic room temperature phosphorescence through rational molecular design
Source: Light Sci Appl. 2022 May 17;11:142. doi: 10.1038/s41377-022-00826-4 (PMC9114335; doi:10.1038/s41377-022-00826-4)
Supplement: Supplementary file 4 — Copyrright for Chart S1-2 [file 41377_2022_826_MOESM4_ESM.pdf]

Help Live Chat

The influence of the molecular packing on the room temperature phosphorescence of purely organic luminogens

Author: Jie Yang et al

Publication: Nature Communications

Publisher: Springer Nature

Date: Feb 26, 2018

Copyright © 2018, The Author(s)

Creative Commons

This is an open access article distributed under the terms of the Creative Commons CC BY license, which permits unrestricted use, distribution, and reproduction in any medium, provided the original work is properly cited.

You are not required to obtain permission to reuse this article.

To request permission for a type of use not listed, please contact Springer Nature

© 2022 Copyright - All Rights Reserved | Copyright Clearance Center, Inc. | Privacy statement | Terms and Conditions

Comments? We would like to hear from you. E-mail us at customercare@copyright.com
